# Biochar suppresses conjugative transfer of antibiotic resistance genes in manure-amended soils

**DOI:** 10.1093/ismejo/wraf187

**Published:** 2025-08-21

**Authors:** Jing Fang, Zhiwen Chen, Zhigang Yu, Shengdao Shan, Yucheng Hou, Lili Liu, Jin Huang, Bing Li, Jianhua Guo

**Affiliations:** School of Environment and Natural Resources, Zhejiang University of Science and Technology, Hangzhou 310023, Zhejiang Province, China; Key Laboratory of Recycling and Eco-treatment of Waste Biomass of Zhejiang Province, Zhejiang University of Science and Technology, Hangzhou 310023, Zhejiang Province, China; School of Environment and Natural Resources, Zhejiang University of Science and Technology, Hangzhou 310023, Zhejiang Province, China; Australian Centre for Water and Environmental Biotechnology, The University of Queensland, St. Lucia, Brisbane, Queensland 4072, Australia; School of Environment and Natural Resources, Zhejiang University of Science and Technology, Hangzhou 310023, Zhejiang Province, China; Key Laboratory of Recycling and Eco-treatment of Waste Biomass of Zhejiang Province, Zhejiang University of Science and Technology, Hangzhou 310023, Zhejiang Province, China; School of Environment and Natural Resources, Zhejiang University of Science and Technology, Hangzhou 310023, Zhejiang Province, China; School of Environment and Natural Resources, Zhejiang University of Science and Technology, Hangzhou 310023, Zhejiang Province, China; Key Laboratory of Microorganism Application and Risk Control, Ministry of Ecology and Environment, Tsinghua Shenzhen International Graduate School, Tsinghua University, Shenzhen 518055, China; School of Minerals Processing and Bioengineering, Central South University, Changsha 410083, Hunan Province, China; Key Laboratory of Microorganism Application and Risk Control, Ministry of Ecology and Environment, Tsinghua Shenzhen International Graduate School, Tsinghua University, Shenzhen 518055, China; Australian Centre for Water and Environmental Biotechnology, The University of Queensland, St. Lucia, Brisbane, Queensland 4072, Australia

**Keywords:** Antibiotic resistance, Conjugative transfer, Biochar, Animal manure, Soil microbiota

## Abstract

The environmental dissemination of antibiotic resistance genes (ARGs), particularly in manure-amended soils, poses a growing threat to public health due to the potential transfer of ARGs to humans and animals. Effective strategies are urgently needed to mitigate ARG spread in agricultural settings. Biochar, an eco-friendly soil amendment, shows promise for pollution control, yet its role in suppressing ARG horizontal gene transfer remains unclear. Here, metagenomic analysis showed that manure application significantly increased the relative abundance of ARGs in soil microbiota, whereas biochar amendment reduced it. To determine whether biochar suppresses ARG dissemination by inhibiting horizontal transfer, we established a soil microcosm. Manure application increased the conjugative transfer ratio by 3-fold, whereas biochar effectively suppressed this transfer—reducing it to levels observed in unamended soils. Cell sorting and 16S rRNA gene amplicon sequencing demonstrated that biochar treatment reduced the diversity of transconjugant pools at both phylum and genus levels. Transconjugants were primarily affiliated with *Pseudomonadota*, *Bacillota*, and *Actinomycetota*, with *Massilia*, *Delftia*, and *Ammoniphilus* being the most abundant genera in biochar treatment soil. Mechanistic investigations revealed that biochar-mediated inhibition of ARG transfer was linked to reduced ATP energy supply, decreased reactive oxygen species production, and lower cell membrane permeability, and diminished bioavailability of heavy metals and antibiotics. Additionally, biochar altered soil enzyme activity and microbial community structure, further limiting ARG dissemination. The findings provide insights into biochar-induced mitigation of ARG spread in manure-amended soils and highlight its potential as an effective strategy for controlling environmental ARG transmission.

## Introduction

With the development of animal husbandry, the global production of livestock and poultry manures continues to rise. The estimated world’s total emissions of animal manures were 9.26 billion tonnes in 2022 alone [1]. Land application of animal manures is a commonly used practice in agriculture and is regarded as a cost-saving way to recycle nutrients from animal wastes [2, 3]. However, the land application of manures has posed ecological security risks [4–6], including but not limited to pathogen contamination, heavy metal pollution, as well as the dissemination of antibiotic resistance genes (ARGs) and antibiotic resistant bacteria (ARB). In particular, the rapid dissemination of ARGs and ARB has been recently recognized as one of the most significant global challenges [7, 8]. An estimate of 4.95 million deaths was associate with antimicrobial resistance in 2019, including 1.27 million deaths directly attributable to ARB-caused infections [8]. Although many deaths associate with ARB occur in clinic settings, there is increasing evidence that environmental reservoirs such as animal husbandry and wastewater are also considered significant contributors [5, 6, 9]. In these environments, ARGs can be maintained or disseminated through: (i) persistence/replication (vertical gene transfer, VGT) of ARG-harboring bacteria; and (ii) horizontal gene transfer (HGT) between bacterial communities [10]. Among the three HGT routes (conjugation, transformation, and transduction), plasmid-mediated conjugative transfer is a dominant way for ARG transmission [10, 11].

Previous studies have shown that the application of manure bears an undeniable responsibility for the dissemination of ARB and ARGs in the environment [12–14]. Manure application could significantly increase the diversity and abundance of ARGs in soil, and also shift soil microbiota [15, 16]. ARG abundance in swine farms was found to be positively correlated with the concentrations of antibiotic and heavy metal derived from farm samples, suggesting the potential of co-selection for resistance traits [13]. Indeed, heavy metal pressure in the environment could facilitate the proliferation of antibiotic resistance via co-selection for ARGs and metal resistance genes [17, 18]. Although previous studies observed the emergence and transfer of ARGs associated with manure application in soils [19–23], the quantification and identification of HGT events are not experimentally evidenced. There is an urgent need to develop a reliable method to reveal HGT dynamics in such complex ecosystems.

It is critical to effectively utilize nutrients in manures but minimizing negative effects associated with manure application. Biochar is an environmentally friendly carbon material, which is formed by the pyrolysis of waste biomass and has been widely used for agricultural soil improvement, pollution remediation, and carbon sequestration [24–26]. Biochar could potentially increase soil water holding and cation exchange capacity, stimulate microbial activity and reduce the nutrient leaching from the root when applied in fields [27]. Biochar could also immobilize and mitigate the contaminant (organic and heavy metal) in soil by sorption, raising soil pH, and modulation of the redox state of soil [28, 29]. For example, a previous study found that combined biochar and manure addition to an agricultural soil could benefit fertility (reducing nutrient P and K leaching), microbial activity, and carbon stabilization [30]. However, the effects of biochar are complicated, as biochar could potentially increase the bioavailability of some toxic metals (e.g. As and Cr) [29], and also release toxic substances that pose a threat to soil organisms [31]. In the past decade, employing biochar to control the dissemination of ARGs in soil has become a research hotspot [32, 33]. Several studies have shown that biochar exhibited a positive effect in reducing the abundance of ARGs in soil by decreasing contaminant bioavailability, altering microbial community structure, inhibiting abundance of mobile genetic elements (MGEs), reducing the selective/co-selective pressure on ARGs, adsorbing and damaging ARGs [34–37]. For example, application of wood biochar reduced the relative abundance of ARGs by around 70% in soil from a swine farm [36]. In contrast, conflicting results were obtained in other studies [38–40]. For example, no significant decrease of ARGs was found in rhizosphere soil after rice straw biochar amendment [39]. Even worse, the manure-derived biochar amendment significantly enriched the ARG abundance [40]. All previous studies only investigated the correlation between the changes in the abundance of ARGs and MGEs in biochar amended soil. However, the causal relationship between biochar and the mitigation of ARGs in soils has not been rigorously elucidated. It remains unclear whether and how biochar could reduce the HGT process of ARGs when simultaneously applied in manured soils. It is also unknown whether biochar could alter the composition of recipient taxa in manured soils.

To address knowledge gaps, our study focuses on ARGs in manure amended soils, specifically those associated with plasmids that facilitate HGT to human and animal pathogens. We hypothesize that biochar could inhibit the conjugative transfer of ARGs in soil, thereby mitigating the dissemination of ARGs in soil. Firstly, we employed metagenomic sequencing to investigate the abundance and diversity of ARGs and MGEs in soils with the treatments of fresh manure and biochar. Next, we constructed a manured soil microcosm amended with biochar to: (i) quantify the plasmid-mediated conjugative transfer ratio in manured soils with or without biochar, (ii) identify the host bacteria carrying plasmid-encoded ARGs in soil microbiota after conjugation, and (iii) explore the mechanisms by which biochar influences ARG conjugation. These mechanisms include changes in transconjugant taxa, bacterial metabolism, soil enzymatic activities, pollutant bioavailability, and microbial community structure. We used *Escherichia coli* MG1655, carrying a broad-host-range plasmid encoding ARGs, as the donor strain and inoculated it into the soil microcosms. Transconjugants were visualized by confocal laser scanning microscope and quantified flow cytometry. Fluorescently labeled transconjugants were then sorted out through fluorescence-activated cell sorting (FACS) and analyzed by 16S rRNA gene amplicon sequencing to characterize the composition of the transconjugant communities with or without biochar amendment. Overall, our findings provide critical insights into the risks of ARG dissemination via manure application and demonstrate the potential of biochar as a practical strategy to mitigate ARG spread in soil ecosystems.

## Materials and methods

### Donor strain

In this study, *E. coli* MG1655 (chromosomally tagged by *lacI^q^-*P*lpp-mCherry*) hosting the broad host range plasmid pKJK5 (IncP1; tagged with P*lac-gfpmut3B*) was used as the donor, which was shared by Dr. Bing Li at University of Science and Technology Beijing (China) and originally donated by Dr Barth F. Smets at Aarhus University, Denmark. The pKJK5 plasmid carries three ARGs, specifically including the trimethoprim (Tmp), tetracycline (Tet), and kanamycin (Km) resistance genes [23]. The donor *E. coli* contain a conjugative plasmid tagged with the green fluorescent protein gene (*gfpmut3B*) downstream from a *LacI* repressible promoter [22, 41]. The donor chromosome encodes *mCherry* red fluorescence and *LacI* that represses the expression of *gfpmut3B*. Hence, the *gfpmut3B* gene only becomes expressed upon successful transferred to a recipient cell (does not encode the *LacI*), resulting in green-fluorescently labeled transconjugant cells. The red-fluorescent donor cell and the green-fluorescent transconjugants were subsequently detected by a confocal laser scanning microscope and flow cytometry [41, 42]. The donor strain was cultured overnight in LB medium supplemented with tetracycline (5 mg/l) and trimethoprim (30 mg/l) to select for plasmid carriage at 37°C under shaking conditions (180 r/min). The minimal inhibitory concentration of tetracycline and trimethoprim for the donor strain ranged from 130–140 mg/l and from 7000–8000 mg/l, respectively (in Supplementary Fig. S1 and Text S1). After culturing, the donor strain was harvested by centrifugation at 8000 *g* for 15 min, washed twice with phosphate-buffered saline (PBS, pH = 7.2) and was resuspended in 0.9% sterile saline solution. The concentration of donor inoculants was measured by colony-forming unit counting.

### Biochar preparation

The prewashed and air-dried wheat straw were pyrolyzed in a muffle furnace under anoxic conditions at a heating rate of 5°C/min to the target pyrolysis temperature of either 300°C or 700°C, which were then held for 2 h. The obtained biochar samples were designated as BC300 and BC700, corresponding to their respective pyrolytic temperatures. In general, pyrolysis at 300°C and 700°C represents low-temperature and high-temperature biochar production, respectively [26]. The biochar samples were ground, sieved through a 100-mesh sieve, and then stored in air-tight containers in a desiccator for later use. The physicochemical characterization of biochar samples, such as C, H, N, O elemental analysis, ash content, pH, and surface areas were described in Supplementary Text S2 and Table S1.

### Set-up of soil microcosms.

Surface soil (0–20 cm) was collected from a vegetable field in Quzhou, China (118°89′2183″E, 29°02′0226”N) on 3 April 2023 and was passed through a 2 mm sieve. The soil was classified as a sandy loam (8.4% clay, 16.3% silt, and 75.3% sand) belonging to red soil, with pH 5.5 and soil organic matter 1.5%. The fresh pig manure was collected from a livestock farm near Hangzhou, China on 2 April 2023, and its pH was 8.2. The soil and manure samples were stored at 4°C and used within 2 weeks to minimize potential changes in the bacterial community structure and activity. To restore the activity of soil microorganisms, soil samples were placed in an incubator for 1–2 h at 30°C. The nutrients (N, P, and K) of soil and manure were determined and described in the Supplementary (Text S3 and Table S2).

To investigate the effect of fresh manure application on the types and abundance of ARGs in soil, we setup soil microcosm series with nonsterilized manure and performed metagenomic analysis. Soil microcosm generally contained 15 g fresh soil, 0.15 g of biochar (about equal to 1% application rate to soil weight), 0.6 g of manure (about equal to 40 mg/g soil), and around 9 ml ultrapure water to form slurry by evenly stirring, and then were aerobically incubated at 30°C in dark condition. The dosages of biochar and manure used in this study belonged to a moderate dose category in the field soil application of biochar [43, 44], and a common low-level dose compared to manure amendments that are typically applied to agricultural soils [45, 46]. The detailed setup method for soil microcosm series with nonsterilized manure was described in Supplementary Text S4.

**Figure 1:**
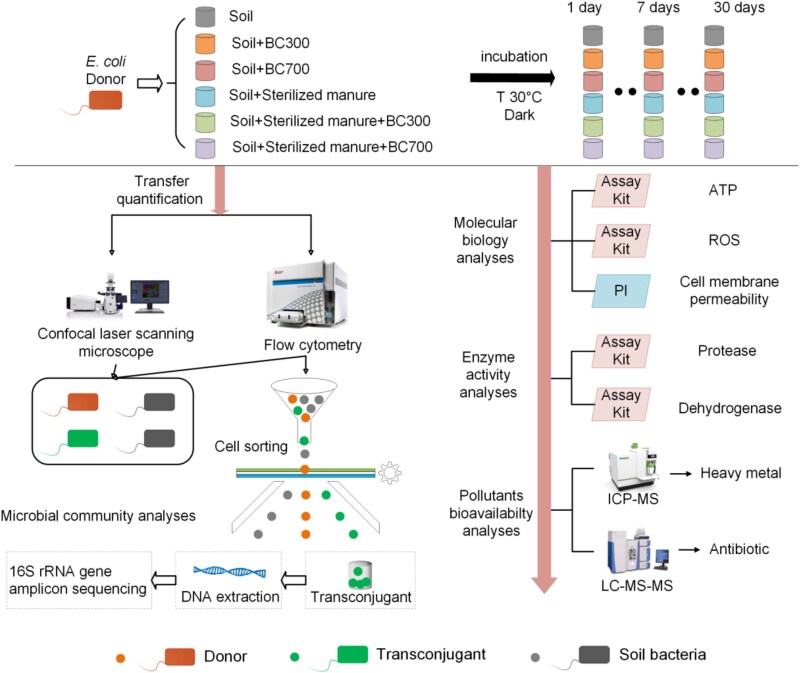
Experimental design and methodologies used in this study. Fluorescently *mCherry*-tagged environmental bacterium *Escherichia coli* MG1655 hosting the broad-host-range plasmid pKJK5 (IncP1; tagged with P*lac*-*gfpmut3B* and contained trimethoprim, tetracycline and kanamycin resistance genes) was added to natural soil microcosms. The expression of *gfpmut3B* was chromosomally repressed in the donor strain, and only expressed upon successfully transferred to soil bacteria strain.

To test whether biochar can inhibit conjugative transfer of ARGs from fecal bacteria to soil bacteria (excluding the transfer between fecal bacteria), we established sterilized manured soil microcosms with the application of biochars (Fig. 1). The manure was sterilized by autoclave before use to eliminate the influence of manure bacteria on the conjugation process and served mainly as nutrient and stress source in the study. The detailed setup method for soil microcosm series with sterilized manure and donor inoculation was described in Supplementary Text S5. Soil microcosms with biochar or manure application were labeled as soil, soil+BC300, soil+BC700, soil+manure, soil+manure+BC300, and soil+manure+BC700 treatment groups. Series microcosms without donor inoculation were also prepared and incubated under the same conditions to investigate the effects of biochar and sterilized manure on the soil microbial community. Soil samples were collected at specific time points of each treatment group, corresponding to days 1, 7, and 30. Samples were partitioned into two subsamples: one stored at 4°C for Nycodenz extraction of bacterial cells and one stored at −20°C for total DNA extraction.

To investigate the impact of pollutants in manure, a soil microcosm with rinsed manure was setup according to the above method. The method for rinsing manure to remove some heavy metals and antibiotics and the corresponding soil microcosms setup was described in Supplementary Text S6. Furthermore, soil microcosms with tetracycline treatment were setup to explore the effect of antibiotics on the conjugative transfer of ARGs in soil and the corresponding process was described detail in Supplementary Text S7. Each of the above soil microcosms were setup with four replicates. In addition, the effect of biochar itself on conjugative transfer rates in pure culture (PBS solution) system was further studied and the corresponding process was described in Supplementary Text S8. Pre-experiment showed that both BC300 and BC700 had no significant effect on the growth of the donor and recipient strains (Supplementary Fig. S2 and Text 9).

### Nycodenz extraction and transconjugants analyses

Nycodenz density gradient separation was used to extract the bacterial communities, as described previously [47], with reagent volumes adapted to match the used 5 g of the soil microcosms. The Nycodenz extracts were stored at 4°C until flow cytometry analysis or cell sorting. Cells obtained from Nycodenz extraction were analyzed and sorted using a Attune NxT flow cytometry (Thermo Fisher Scientific) and BD FACS melody 488 (BD Biosciences), respectively. Detailed analysis and sorting methods of transconjugants were presented in the Supplementary (Text S10). A minimum of 50 000 cells were acquired in all sorting runs for subsequent DNA extraction and 16S rRNA gene amplicon sequencing. In addition, transconjugants and donor bacteria were visualized by a confocal laser scanning microscope (CLSM) (LSM900, Zeiss, Germany) with excitation at 488 nm (FITC) and 561 nm (*mCherry*), respectively. The transfer ratio was calculated as the number of transconjugants divided by the total detected cells per measurement (1 × 10^6^ cells).

### DNA extractions and 16S rRNA gene amplicon sequencing analysis

All DNA extractions were performed with the FastDNA Spin kit for soil (MP Biomedical, Santa Ana, CA, United States) following the manufacturer’s instructions. Soil DNA was extracted from 0.5 g of soil, and the DNA from the transconjugants was obtained after concentrating the cells by centrifugation (10 000 *g*) and resuspension in 250 μL of sterile PBS. 16S rRNA gene amplicon sequencing was conducted at Majorbio Testing Center (Shanghai Meiji Biomedical Technology Co., Ltd). Details of 16S rRNA gene amplicon sequencing and bioinformatics analysis methods were described in Supplementary Text S11. All samples were prepared in biological duplicate.

### Identification of ARGs and MGEs

Metagenomic sequencing and the corresponding analysis techniques were used to identify ARGs and MGEs in soil with the treatments of manure and biochar. The standard pipeline of ARGs-OAP, along with the SARG database (version 3.2), was employed to assess the abundance of ARGs in each sample using the clean paired-end reads (Q > 20) [48]. A sequence was classified as an ARG-like sequence if it satisfied the alignment criteria of ≥80% similarity and a minimum alignment length of 38 amino acids (≥75% of the read length), as recommended [49]. Additionally, MGEs were identified by aligning the clean reads against the mobileOG-db, a manually curated MGE database, using USEARCH [50]. For further precise classification, the Basic Local Alignment Search Tool (BLAST) was utilized, with a cutoff set at ≥80% identity and ≥75% coverage. The abundance of ARGs and MGEs was normalized and ultimately expressed as “Ratio of ARG to 16S rRNA gene copies” [51], thus enabling to compare each other between different treatment groups.

### Measurement of adenosine triphosphate (ATP), reactive oxygen species (ROS), and cell membrane permeability of soil bacteria

Changes in ATP level, intracellular ROS production, and cell membrane permeability of the overall soil bacteria were measured for various treatment groups in soil microcosm. Bacteria from soil microcosm samples were extracted by Nycodenz density gradient separation and were resuspended in PBS. According to previous studies [42, 52], the cellular ATP was determined using an enhanced ATP Assay Kit (Beyotime, China) according to the instructions and finally detected with a chemiluminescence instrument (Varioskan LUX, Thermo Scientific). The intracellular ROS was detected using a ROS Assay Kit (Beyotime, China), namely the fluorescent probe DCFH-DA. The suspensions were analyzed with a multimode reader (Varioskan LUX, Thermo Scientific) with excitation at 488 nm and emission at 525 nm. The cell membrane permeability was determined by the DNA-intercalating fluorescent dye propidium iodide (PI) [53]. The suspensions were analyzed by a multimode reader (Varioskan LUX, Thermo Fisher Scientific) with excitation at 535 nm and emission at 615 nm. All samples were prepared in biological triplicates and technical duplicates.

### Antibiotic and heavy metal quantitation in soil microcosms

Given that the bioavailability of antibiotics and heavy metals, rather than the total amount in soil is the most important, we mainly focus on the bioavailability of pollutants, such as water-soluble antibiotics and heavy metals extracted from DTPA-TEA-CaCl_2_ [54]. Sample extraction and analysis procedures for antibiotics and heavy metals were described in Supplementary Text S12. The initial contents of antibiotics and heavy metals in the manure were shown in Supplementary Tables S3–S4.

### Determination of soil enzyme activity

Soil enzymes, such as protease and dehydrogenase, were determined using soil ELISA kits and soil dehydrogenase (S-DHA) kits [55], respectively. Soil enzyme activity was measured on a multimode reader (Varioskan LUX, Thermo Scientific) at 450 nm.

### Statistical analysis

All phenotypic data were expressed as mean ± standard deviation. SPSS for Mac version 25.0 was applied for data analysis. Independent-sample *t* test was performed, and the significant differences were analyzed using one way ANOVA with Tukey HSD posthoc test. *P* values less than 0.05 were considered significant differences.

## Results and discussion

### Characteristics of biochar and soil microcosm

Two types of biochar were alkaline, with pH of 8.1 and 10.8 for BC300 and BC700, respectively (Supplementary Table S1). BC700 had higher surface areas, ash, and C content, whereas BC300 had more O content and H/C, O/C, and (O + N)/C ratios. These results were consistent with previous studies [56, 57], in which a large amount of O element and polar functional groups in biochar lost, as stable aromatic carbon structures formed with the increase of pyrolysis temperature. The physicochemical characteristics of biochar can substantially influence the soil composition, especially soil pH [58]. The pH of the soil microcosm with manure was increased from 5.5 to neutral of 6.9 (Supplementary Table S2). The simultaneous application of biochar and manure in the soil microcosm further increased the pH, which was 7.2 and 7.7 after treated with BC300 and BC700, respectively. Meanwhile, biochar also increased the availability of nutrients (N, P, and K) in soil microcosms (Supplementary Table S2). As indicated by previous studies, increase in soil pH and nutrients significantly impacted on microbial communities, enzyme activity, and the bioavailability of heavy metals [44, 59, 60], which may relate to the spread of ARGs in soils.

### Identification of ARGs and MGEs in soil microcosms by metagenomics techniques

**Figure 2:**
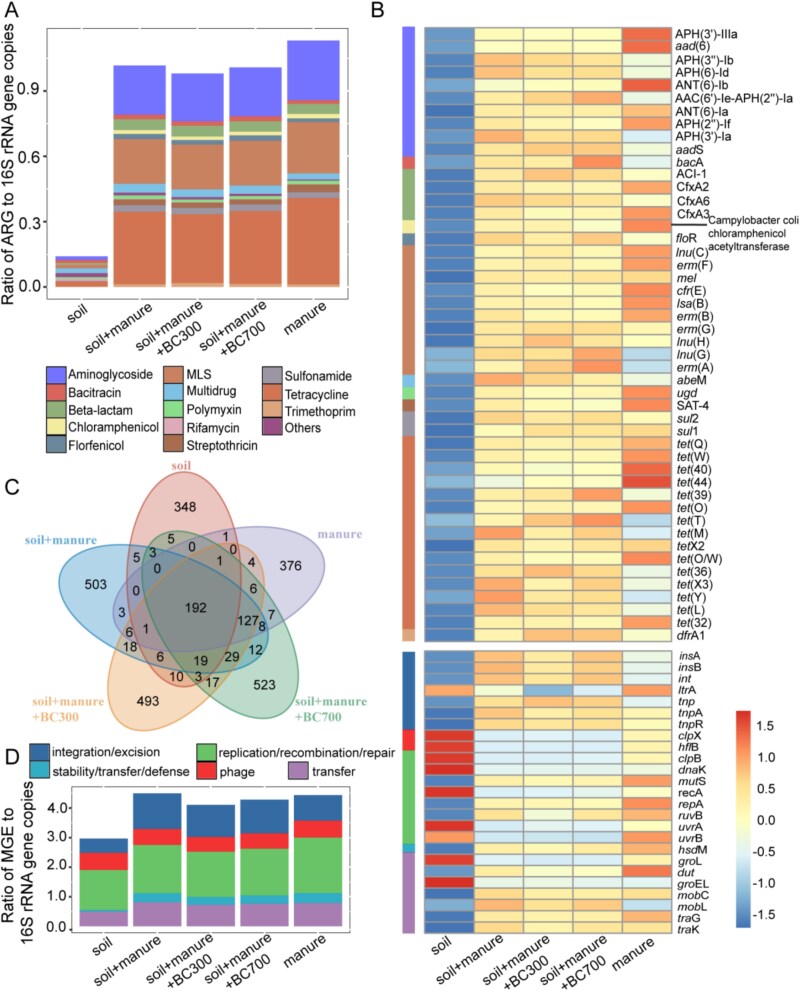
Bioinformatics analysis for quantifying ARGs and MGEs according to metagenomic data in different soil microcosms. (A) Broad-spectrum quantitative profile of the ARG types (ratio of ARG to 16S rRNA gene copies); (B) Abundance of the major ARG and MGE subtypes (the ratio greater than 0.005 in any sample), color scale represents the relative abundance of ARGs after Z-score normalization; (C) Venn diagram of the number of ARGs identified. (D) Broad-spectrum quantitative profile of the MGE types (ratio of MGE to 16S rRNA gene copies). The manure referred above was nonsterilized (original) manure.

The profiles of ARGs and MGEs after the application of manure or biochar in soil were revealed through metagenomic sequencing. In total, 14 types of ARGs and more than 594 ARG subtypes were detected in at least one of the samples (Fig. 2A). Manure samples harbored the highest abundances of ARGs, whereas the soil sample without treatment had the lowest. After fertilization with manure, the relative abundances of ARGs in soil increased by 6.2 times, and the vast majority of ARG subtypes were positively correlated with ARG subtypes in manure (Fig. 2B, *P* < 0.05), indicating the spread of ARGs from manure to soil. The application of manure also changed the structure of ARGs in the soil (Fig. 2C), although the application rate of manure in soil was only 4% (40 mg/g). The ARGs harbored by manure should be diluted after entering the soil. However, their abundance was only decreased by 10.1%. There are two possible reasons: (i) ARB in manure reproduce in the soil and ARGs spread by VGT; and (ii) ARB in manure transfer their ARGs to indigenous bacteria in soil through HGT. Specifically, several bacterial phyla increased their relative abundances in the manured soil (Supplementary Fig. S3), including *Bacillota, Bacteroidota, Spirochaetota*, *Uroviricota, Tenericute,* and *Fibrobacterota*, which had extremely low abundances in the control soil. This trend indicates that fecal bacteria potentially reproduce themselves in the soil. The current study also observed that the abundance of MGEs in soil samples was much higher than (increased by 51.6%) that in the soil itself after the treatment of manure (Fig. 2D), indicating the HGT potentially occurred in the manured soil. Indeed, HGT has been widely acknowledged as an important pathway, based on substantial survey data showing a positive correlation between the abundance of ARGs and MGEs [6, 13, 39].

The amendment of biochar reduced the abundance of ARGs by 0.9–3.6% and MGEs by 4.8–8.7% in the soil treated with manure (Fig. 2A, D). Genes encoding resistance against aminoglycoside, tetracycline, multidrug, streptothricin, chloramphenicol, polymyxin, and rifamycin were decreased due to the treatment of biochar. Among them, ARG subtypes of *tet(M)*, *tet(X3)*, *tet(Y)*, *tet(L)*, *APH(3″)-Ib*, *APH(6)-Id*, *APH(3′)-Ia*, *CfxA6,* and *abeM* showed the most significant decrease (Fig. 2B). Furthermore, biochar also altered the abundance of certain bacterial communities in soil treated with manure (Supplementary Fig. S3). These results suggest that the addition of biochar potentially affect both the VGT process of ARB and the HGT process of ARGs in manured soil. The following experiments aimed to reveal whether and how the application biochar suppresses the HGT process of ARGs from manure to soil bacteria.

### Biochar suppresses conjugative transfer of ARGs in soil

**Figure 3:**
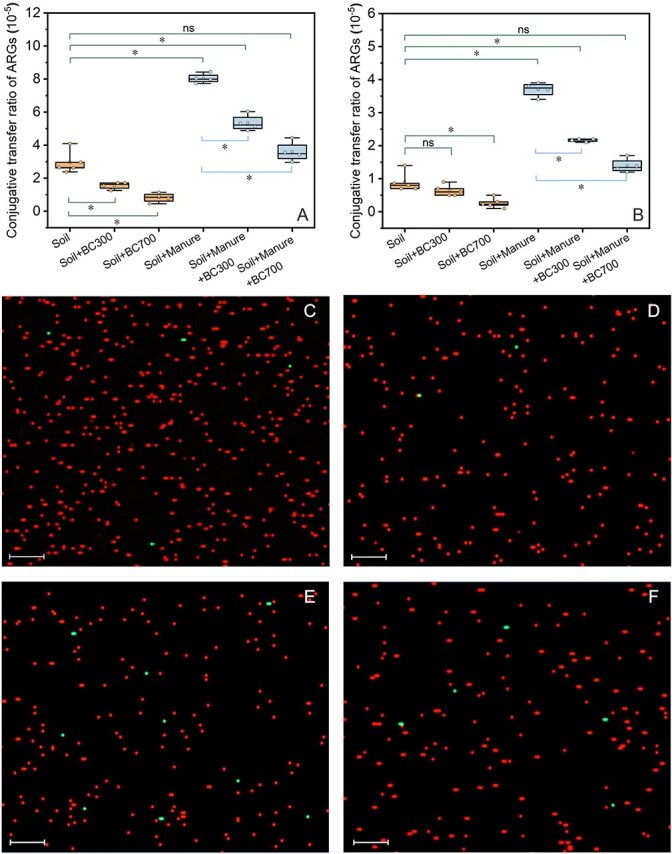
Changes of transconjugants following the application of biochar in soil microcosms. (A) Incubation for 1 day, (B) Incubation for 7 days. The significant differences between different treatments are indicated with ^*^P < 0.05, and ns indicates no significant difference. Confocal laser scanning microscope (CLSM) images of the donor bacteria (red fluorescence) and transconjugants (green fluorescence) after incubation for 1 day. (C) Soil, (D) Soil+BC700, (E) Soil+manure, (F) Soil+manure+BC700. The manure referred above was sterilized manure. The original CLSM images were enhanced and adjusted for color contrast by ZEISS ZEN 3.12 application program. Scale bars = 8.5 μm.

For both nonmanured and manured soil microcosms, the application of 1% biochar significantly reduced conjugative transfer ratio of ARGs (Fig. 3, *P* < 0.05). After 1 day incubation, the conjugative transfer ratio was observed at (2.94 ± 0.62) × 10^−5^ for the control soil microcosm (Fig. 3A). This level is comparable to the one from previous soil studies [23, 41, 61]. The transfer ratio decreased to (1.55 ± 0.21) × 10^−5^ and (0.82 ± 0.24) × 10^−5^ after treatment with BC300 and BC700 in nonmanured soil microcosms, respectively. After the application of sterilized manure, the conjugative transfer ratio was (8.04 ± 0.22) × 10^−5^, which was around 3-fold that of the nonamended control soil. Such transfer ratio was declined to (5.34 ± 0.41) × 10^−5^ and (3.59 ± 0.52) × 10^−5^, when simultaneously applying sterilized manure with BC300 and BC700, respectively, with the descender of 33.6% and 55.3% compared to the only manure treatment. Although conjugative transfer ratio in manure with BC300 treatment was still significantly (*P* < 0.05) higher than the control soil group, this ratio in the manure with BC700 treatment was already very close to that of the control soil with no significant difference (*P* > 0.05). As well, the number of visible green-fluorescent transconjugants in biochar treated groups was much less than those without biochar (Figs. 3C–F, Supplementary Fig. S4).

After 7-day incubation (Fig. 3B), the results followed the same trend to those after incubation for 1 day. The conjugative transfer ratio of sterilized manure treatment was still significantly higher than that of the control soil (*P* < 0.05), which was four times higher with the ratio of (3.69 ± 0.22) × 10^−5^. However, the application of biochar resulted in 41.3–62.3% decrease of conjugative transfer ratio in manured soil. We noted that the conjugative transfer ratio naturally decreased with the extension of cultivation time, regardless of biochar treatment. For example, on day 7, conjugative transfer ratio of the control soil group was dropped by 69% compared to day 1 (Fig. 3A and B). At the same time, the number of donor *E. coli* also declined to less than 0.1% of that on day 1 (Supplementary Table S5). Similar trend was observed on day 30, as we just detected 1–2 donor cells and did not detect any transconjugants per measurement (Supplementary Table S6). These results indicate that it is difficult for transconjugants and donor bacteria to colonize in actual soil environments, which may be linked to the loss of plasmids pKJK5. It also suggests that other factors may play a role in hampering the colonization of the plasmids in new hosts, e.g. fitness cost, soil bacterial community composition, nutrient competition, and selective pressures [20–23, 41].

### Biochar alters the transconjugant pools in manured soil

**Figure 4:**
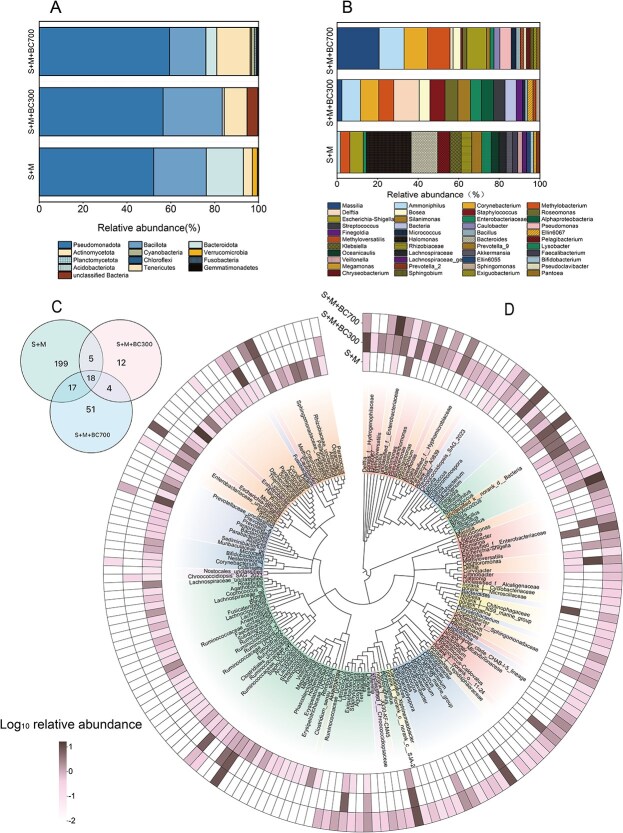
Bacterial community compositions in transconjugant pools of soil microcosms. (A) Transconjugant microbial community composition at phylum level. (B) Transconjugant microbial community composition at genus level. (C) Venn diagram of the number of genera identified. (D) Phylogenetic trees were constructed using the sequencing of OTUs with relative abundance more than 0.1% in the transconjugant pools. S and M represents the soil and sterilized manure, respectively.

The manure used in this study was sterilized, and we assumed that all the genera of transconjugants detected were derived from soil bacteria. We found that biochar altered the composition of transconjugants pools in soils at both phylum and genus levels (Fig. 4A, Supplementary Table S7). In manured soil microcosms without biochar, totally 16 phyla were identified in the transconjugant microbial community. *Pseudomonadota* was the most dominant phylum, contributing to 52.1% of the transconjugant pools. It was followed by *Bacillota* (23.9%), *Bacteroidota* (16.8%), *Actinomycetota* (4.1%), *Verrucomicrobiota* (2.3%), *Cyanobacteria* (0.2%), and *Fusobacteriota* (0.1%). The top four phyla bacteria that were able to acquire the conjugative plasmid in soil have been documented in previous studies [21–23].

In the presence of biochar, less phyla were identified in the transconjugants pools of soil (Fig. 4A, Supplementary Table S7). With the application of BC300, there were totally six phyla identified in the transconjugant microbial community. *Pseudomonadota* was again the most dominant phylum, contributing 56.4%, followed by *Bacillota* (27.0%), *Actinomycetota* (10.4%), *unclassified Bacteria* (4.9%), *Bacteroidota* (0.9%), and *Cyanobacteria* (0.3%). By the application of BC700, 13 phyla were identified in the transconjugant microbial community, ranking as *Pseudomonadota* (59.3%), *Bacillota* (16.5%), *Actinomycetota* (15.2%), *Bacteroidota* (4.9%), *Cyanobacteria* (1.5%), *Planctomycetota* (0.7%), *Chloroflexi* (0.6%), *Verrucomicrobiota* (0.5%), *Fusobacteriota* (0.3%), and *Acidobacteriota* (0.2%). Biochar not only reduced the transconjugant bacteria phyla in soil, but also changed the relative abundance of bacteria. Biochar decreased the relative abundance of *Bacteroidota* (less than 5%) and increased the *Actinomycetota* (exceeding 10%) in transconjugants pools. In the presence of biochar, four phyla bacteria including *Tenericutes*, *Gemmatimonadetes*, *Deinococcus-Thermus,* and *WPS-2* disappeared from the transconjugants pools. Instead, two new ARGs recipient phyla *Bdellovibrionota* and *Nitrospirota* were found in manured soil with BC700, though their relative abundance was only 0.04%.

Compared to the phylum level, changes in the genus level caused by biochar are more obvious in the transconjugant community (Fig. 4B). In the manured soil microcosm without biochar, totally 239 genera were identified in the transconjugant microbial community (Supplementary Table S7), of which the top 5 transconjugant genera in abundance ranked as *Halomonas* (16.5%), *Bacteroides* (9.7%), *Escherichia-Shigella* (4.9%), *Pelagibacterium* (4.5%), and *Klebsiella* (4.2%). Among them, four genera are affiliated to *Pseudomonadota* and one genus is affiliated to *Bacteroidota*. In addition to *Escherichia-Shigella* and *Klebsiella* with high pathogenic potential, several opportunistic pathogens were also identified in the manured soil transconjugant pool (Supplementary Table S7), such as *Acinetobacter*, *Pseudomonas*, *Staphylococcus*, *Bacillus*, *Clostridium*, *Cutibacterium*, *Haemophilus*, *Enterococcus,* and *Corynebacterium* [62, 63].

In the presence of biochar, much less genera were identified in the transconjugants pools, which had 39 and 90 genera for BC300 and BC700 application, respectively (Supplementary Table S7). In manured soil with BC300, the top 5 transconjugant genera in abundance ranked: *Delftia* (11.3%), *Ammoniphilus* (8.2%), *Corynebacterium* (8.1%), *Methylobacterium* (6.7%), and *Staphylococcus* (6.7%), which belong to the phyla of *Pseudomonadota, Bacillota,* and *Actinobacteriota*. In manured soil with BC700, the top 5 transconjugant genera in abundance ranked: *Massilia* (15.7%), *Ammoniphilus* (9.3%), *Corynebacterium* (8.7%), *Methylobacterium* (8.4%), and *E. Shigella* (7.4%), which also belonged to the phyla of *Pseudomonadota, Bacillota,* and *Actinobacteriota*. All the three transconjugant pools in soil microcosms shared 18 genera (Fig. 4C), and some genera were only identified in one of the transconjugant pools, especially the manured soil without biochar treatment (with 199 unique genera). The shared genera included pathogens and opportunistic pathogens such as *Staphylococcus*, *Bacillus*, *Cutibacterium*, *Corynebacterium,* and *Klebsiella*. Nonetheless, the application of biochar had reduced the species of pathogens and opportunistic pathogens in the manured soil transconjugant pools (Supplementary Table S7).

Phylogenetic trees were constructed using the sequencing of OTUs with relative abundance more than 0.1% in the transconjugant pools (Fig. 4D). The data show that the components of the main bacterial genera in the three transconjugant pools were significantly different. Plasmid pKJK5 was able to transfer from the donor *E. coli* to a broad range of recipients, with *Pseudomonadota* accounted for the dominant phyla. This can be expected because the donor was relatively phylogenetically close to *Pseudomonadota*, which may favor the conjugative plasmid transfer [64]*.*

### Biochar decreases the ATP, ROS production, and cell membrane permeability of soil microbiome

**Figure 5:**
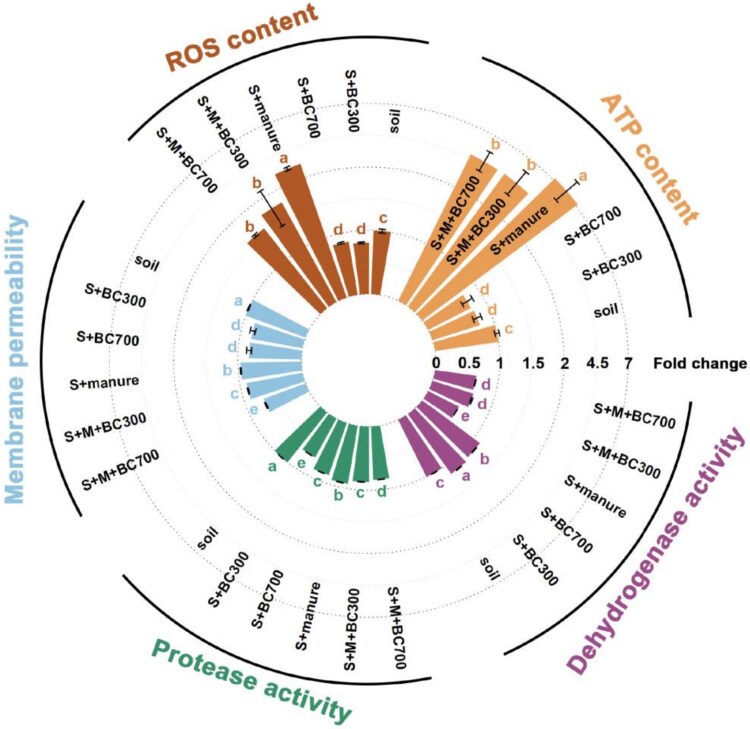
Effects of biochar on the energy supply (ATP), intracellular ROS content, membrane permeability of soil bacteria, the soil protease and dehydrogenase activity in soil microcosms (after incubation for 1 day). All values are expressed as fold changes to the control soil group. S and M represents the soil and sterilized manure, respectively. Under the same indicator, different lowercase letters represent significant differences between treatment groups at the level of *P* < 0.05.

Plasmid-mediated conjugation is an energy consuming process [65]. To explore whether biochar could affect the energy supply of soil bacteria, we monitored the ATP production in the whole soil microbiome for both non manured and manured soil microcosms. Compared to the non manured soil, applying manure obviously increased the ATP production level in soil microbiome by six times (Fig. 5). This is because manure could provide more nutrients for soil bacteria to synthesize energy (Supplementary Table S2). We further found that biochar significantly (*P* < 0.05) reduced soil bacteria ATP production in both non manured and manured soil microcosms by ~24.3% and 35.0%, respectively. Consequently, the reduction of ATP supply by biochar may contribute to the lower level of the conjugative transfer efficiency in soils.

ROS are natural byproducts of bacterial metabolisms, and their production increases under environmental stress like antibiotics, heavy metals, by “SOS response”, playing a role in enhancing conjugative transfer [66–68]. Compared to the control soil, applying manure significantly increased the ROS production level of soil bacteria by 1.2 times (Fig. 5). The high content of antibiotics and heavy metals in the manure from an intensive livestock farm used here may have caused the stress on soil bacteria (Supplementary Table S3–S4), resulting in an increase in ROS. In contrast, the treatment with biochar decreased bacterial ROS levels by ~23.9% for the manured soil microcosms (Fig. 5). This indicates that biochar enables to mitigate the stress of pollutants from manure posed on soil bacteria. Biochar indeed reduced the bioavailability of heavy metals and antibiotics in manured soil, which was elaborated in the later section.

Considring that bacterial membranes are barriers to the conjugative transfer of ARGs, the reduction permeability of which could inhibit conjugative transfer process [69]. Compared to the control groups, biochar significantly (*P* < 0.05) decreased the soil bacteria membrane permeability by ~17.5% and 6.7–26.6% in non manured and manured soil, respectively (Fig. 5). Previous studies also revealed that biochar, especially the biochar from a higher pyrolysis temperature (500°C and 700°C) could reduce cell membrane permeability of *E. coli* [35, 52, 70]. Gene expression involved in outer membrane permeability could be down-regulated with biochar treatment [52], which resulted in decreasing plasmid of ARG-encoded mobilization to soil bacteria.

### Biochar alters enzyme activity level in soil

Enzyme activity in soils is commonly regarded as an important indicator of soil microbial metabolic activity, e.g. microbial conversion of nutrients and detoxification of exogenous compounds [71, 72]. Protease plays an important role in N mineralization in soil, which is related to the absorption of nutrients and energy supply for microbial community [73]. Dehydrogenase is a common redox enzyme, and its activity is an important parameter for measuring pollutant stress on microbial metabolic activity [55]. We found that compared to the control groups (non manured and manured soils), the addition of biochar significantly reduced the activity of soil protease by 13.4–30.5% and 5.5–9.2% respectively (Fig. 5). This reduction in protease activity may hinder the mineralization of organic matter and energy supply of microorganisms. In contrast, the activity of dehydrogenase was significantly (*P* < 0.05) enhanced with the application of biochar (Fig. 5), indicating that environmental stress on soil microbiome can be alleviated by biochar.

### Biochar reduces the bioavailability of manure pollutants in soil

Antibiotics and heavy metals are important stress factors promoting horizontal transfer of ARGs [13, 17],, and pig manure from intensive livestock farm often contains a large amount of these two types of pollutants [74]. Biochar is an excellent type of adsorbent with good adsorption efficiency for various heavy metals and organic compounds, thus reducing their mobility in soil [29]. We speculated that biochar could reduce the bioavailability of these selective compounds in soil, thereby weakening their co-selective pressure on conjugative transfer of ARGs. To this end, we tested the bioavailability of five commonly detected heavy metals and three antibiotics in both non manured and manured soil microcosms.

**Figure 6:**
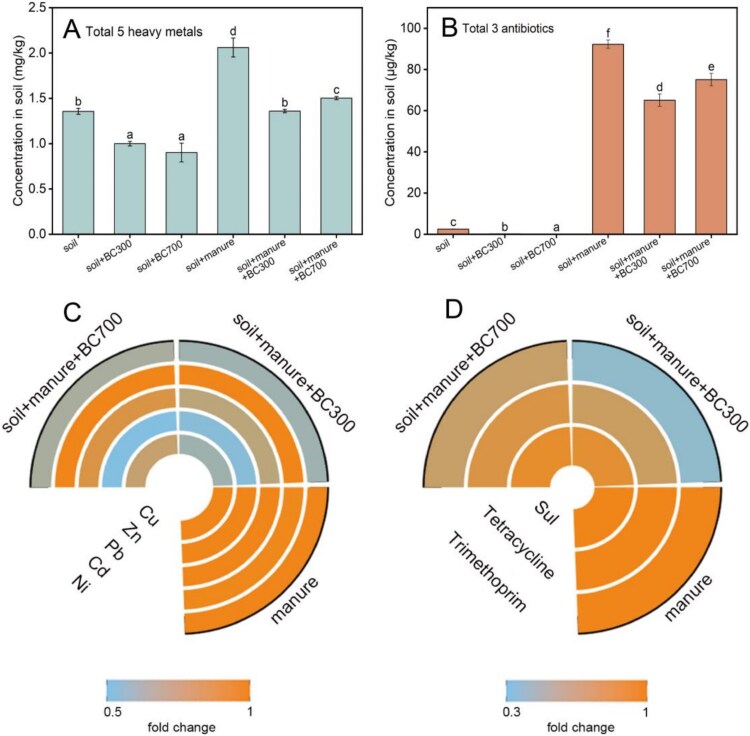
The bioavailable heavy metals and antibiotics in soil microcosms after incubation for 1 day. (A) Total bioavailable heavy metals; (B) Total bioavailable antibiotics; C: Fold changes of Cu, Zn, Pb, Cd and Ni in manured soil microcosms; (D) Fold changes of sulphacetamide (Sul), tetracycline and trimethoprim in manured soil microcosms. Under the same indicator, different lowercase letters represent significant differences between treatment groups at the level of *P* < 0.05. The manure referred above was sterilized manure.

As expected, the bioavailable levels of both heavy metals and antibiotics in the control soil was very low, but they were increased by 50% and 35 times after applying sterilized manure (2.1 mg/kg of heavy metals, 92.2 μg/kg of antibiotics), respectively (Fig. 6; Supplementary Fig. S5). This suggests a serious pollution of heavy metals and antibiotics in soil amended with manure. However, the application of biochar significantly (*P* < 0.05) reduced the bioavailable contents of Cu, Zn, Pb, and Ni in both non manured and manured soil microcosms, with the total bioavailable concentrations of heavy metals reduced by 26.3–33.4% and 27.1–34.6%, respectively (Fig. 6A, C; Supplementary Fig. S5A–E). A similar trend was also observed for the bioavailable content of antibiotics in soil microcosms, especially for tetracycline and trimethoprim, with the total bioavailable concentrations of antibiotics was reduced by 18.6–29.3% after biochar amendment (Fig. 6B, D; Supplementary Fig. S5F–H).

**Figure 7:**
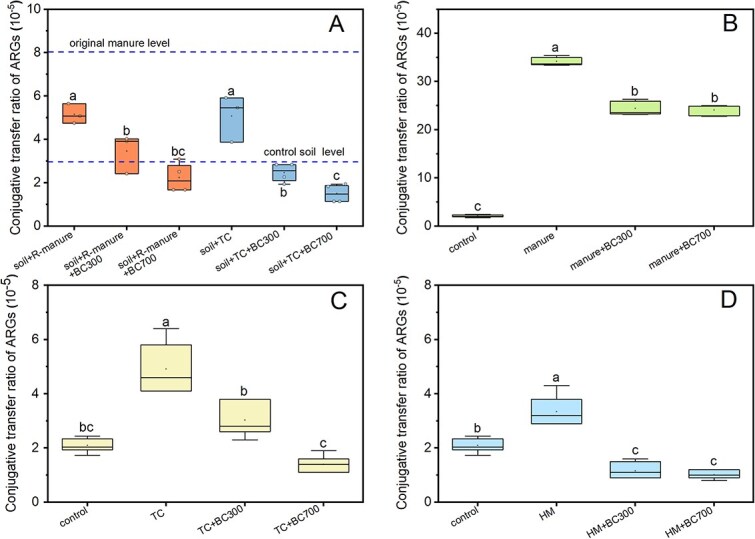
Changes of transconjugants following application of biochar in soil microcosms with or without rinsed manure (R-manure) and tetracycline (TC) (A) and changes of transconjugants following the application of biochar in various phosphate-buffered saline (PBS) solutions (B–D). (B) with manure (10 mg/ ml) in PBS; (C) with tetracycline (TC, 50 μg/L) in PBS; (D) with heavy metals (HM, Cu 50 μg/L + Zn 92 μg/L + Pb 11 μg/L + Cd 10 μg/L + Ni 18 μg/L) in PBS. S represents soil. The manure referred above was sterilized manure. The addition dosage of TC was 5 mg/kg soil. Under the same subplot, different lowercase letters represent significant differences between treatment groups at the level of *P* < 0.05.

To confirm the effect of manure pollutants on conjugative transfer of ARGs in soil, we rinsed the manure and removed over 70% of heavy metals and 79% of antibiotics (Supplementary Tables S3–S4). In soil microcosms with application of rinsed-manure, the conjugative transfer ratio was (5.16 ± 0.36) × 10^−5^ (Fig. 7A), which was significantly (*P* < 0.05) lower than the group (8.04 ± 0.22) × 10^−5^ with original manure. Similarly, adding biochar to soil microcosms with the rinsed-manure significantly reduced its conjugative transfer ratio to (3.45 ± 0.73) × 10^−5^ and (2.23 ± 0.61) × 10^−5^ with BC300 and BC700, respectively, which were very close to the control soil level. Moreover, we investigated the conjugative efficiency in the soil microcosms with the addition of tetracycline. It was found that the conjugative transfer ratio of tetracycline treatment group was (5.08 ± 0.87) × 10^−5^ (Fig. 7A), which was significantly (*P* < 0.05) higher than the control soil group (2.94 ± 0.62) × 10^−5^. When applied biochar (both BC300 and BC700), the conjugative transfer ratio was again significantly reduced to the control soil level, or even lower (*P* < 0.05).

We further investigated the effect of biochar itself on conjugative transfer rates in pure culture models. We found that the manure treatment significantly increased the conjugative transfer ratio up to 15 times, compared to the control group (Fig. 7B, *P* < 0.05). In contrast, the conjugative transfer ratio significantly (*P* < 0.05) declined after applying BC300 or BC700. Moreover, exposures to antibiotics or heavy metals with an equivalent concentration to their level in the manure also significantly increased the conjugative transfer ratio of ARGs (*P* < 0.05), whereas the addition of biochar decreased the transfer ratio to the control level (Fig. 7C, D).

### Biochar alters soil microbial community structure

To evaluate the effects of biochar on microbial communities in soil, the diversity indexes of the soil microcosms were calculated. Overall, the number of classified OTUs in sterilized manured soil microcosms was much less than those in nonmanured soils (Supplementary Fig. S6) and correspondingly the Chao1 index was significantly (*P* < 0.05) lower in the manured soil microcosms (Supplementary Figs. S7–S9), indicating that the application of sterilized manure reduced the soil microbial diversity. This reduction in soil microbial diversity could be due to manure chemical compositions and toxic matters (e.g. heavy metals and antibiotics) [75].

After one day of cultivation (Supplementary Fig. S7), individual manure application enabled *Bacillota* bacteria to be dominant (94.6%) group in soil. The simultaneous application of biochar significantly promoted the growth of *Pseudomonadota*, making both *Bacillota* and *Pseudomonadota* as dominant bacteria. Previous studies have also found that biochar could reduce the abundance of *Bacillota* bacteria in soil or composting systems [37, 38]. At genus level, bacterial components in the manured soil microcosms were markedly different from those in the control soil, in which 9 of the top 10 genera of bacteria belonged to the *Bacillota*. In contrast, with the addition of biochar, 3 of the top 10 genera were affiliated with *Pseudomonadota* (Supplementary Table S8). Biochar-induced alterations in microbial community distribution were closely related to soil properties and biochar types that suppress or increase the specific microbial community [76]. The alkaline nature of the biochar increased the soil pH (Supplementary Table S2), which would lead to more abundance of *Pseudomonadota* and *Actinomycetota* at a higher pH condition [44, 77]. As the cultivation time prolonged to 7 and 30 days (Supplementary Fig. S8, S9, Tables S9, S10), microbial diversity (Chao1 index) in the manured soil increased, although it was still significantly lower than the control soil. Biochar continuously regulated the microbial community structure in soil, leading to an increase in soil microbial diversity. Previous global meta-analysis also revealed positive effects of biochar on soil microbial diversity [78].

### Mechanistic underlying biochar-mediated inhibition of ARG transfer

**Figure 8:**
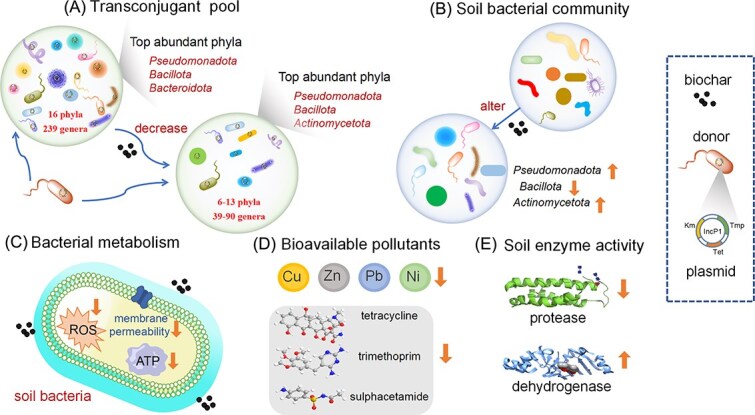
Underlying mechanisms related to the inhibition effect of biochar on conjugative ARG transfer process in manured soils. (A) Biochar decreases the diversity of transconjugant pools in soil; (B) biochar alters the soil microbial community; (C) biochar reduces the production of ATP and ROS, and cell membrane permeability of soil bacteria; (D) biochar decreases the bioavailability of pollutants; (E) biochar alters soil protease and dehydrogenase activity.

In this study, we confirmed that the application of fresh manure significantly increased the abundance of ARGs and MGEs in soil using metagenomic analysis. We further validated that the application of manure significantly promoted plasmid-mediated horizontal transfer of ARGs through soil microcosm experiments. From the perspectives of transconjugant communities, bacterial metabolism, soil enzyme activity, pollutant bioavailability and soil bacteria community, we proposed the underlying mechanisms related to the biochar-mediated inhibition on ARG conjugative transfer in manured soil within 1 day (Fig. 8). The effect of biochar on the conjugative transfer of ARGs in soils is multifaceted, including the altered transconjugant bacteria taxa in soil, declines in ATP energy supply, ROS production, and cell membrane permeability of soil bacteria, reduction in soil protease and increased dehydrogenase activities, reduction in the bioavailability of heavy metals and antibiotics in soil, and the regulation of soil microbial community structure.

We identified the changes of biochar on the transconjugant pools in manured soil. Previous studies mostly speculated on the role of biochar in horizontal transfer of ARGs through changes in MGE and correlation analysis [17, 37, 38, 79]. Our results demonstrated that the application of biochar could decrease the diversity of transconjugants at both phylum and genus levels. Compared to the manured soil without biochar, the number of transconjugants phyla and genera declined by 62.5% and 83.7% for the application of BC300, respectively. Similarly, they declined by 18.7% and 62.3% for BC700 application. Biochar altered the composition of transconjugants pools by increasing the relative abundance of *Actinomycetota* but decreasing *Bacteroidota.* This resulted in *Actinomycetota* as the third abundant transconjugant phylum following *Pseudomonadota* and *Bacillota*. This study indicates that biochar can potentially suppress the conjugative transfer of ARGs across some bacterial taxa in soil, which may contribute to reducing HGT efficiency. In addition, we found a significant difference in the transconjugant pools between soil microcosms with BC300 and BC700, especially at genus level (Fig. 4D), which might be attributed to the properties of biochars and their effects on the soil. The changes in soil pH and nutrient levels play a crucial role in shaping the abundance and activity of microbial communities [59, 80]. BC300 had a higher oxygen content than BC700 (Supplementary Table S1), indicating a higher surface hydrophilicity [56]. BC700 was more alkaline than BC300, which made the soil pH become more alkaline (Supplementary Table S2). Available N and K in the manured soil with BC300 was lower than those with BC700, and the Olsen-P was higher. Correspondingly, BC700 promoted the abundance of *Pseudomonadota* and *Actinomycetota* more effectively than BC300 in manured soil (Supplementary Fig. S7). However, it is still unclear how the pyrolysis temperature of biochar affects the soil transconjugant pools of ARGs, which warrants further research.

ATP is the universal energy carrier for bacterial growth and conjugative transfer process. The reduction of bacterial ATP production by biochar could lead to the insufficient energy supply for conjugative transfer. Form tricarboxylic acid cycle (TCA cycle) related gene expression, biochar was proved to inhibit the gene expression related to Cyt C and complex IV pathway [52]. ROS production is considered a critical index for the conjugative transfer of ARGs [81]. Exogenous substances, such as antibiotics, heavy metals, nonantibiotics pharmaceuticals, and artificial sweeteners can cause an increase in bacterial ROS, triggering the stress-response in bacteria, thus enhancing the conjugative transfer [22, 81–84]. We found that biochar could significantly alleviate such stress posed on bacteria by significantly reducing ROS levels, consequently contributing to the decrease of conjugative transfer in manured soil. In addition to ATP and ROS, cell membrane permeability is also commonly considered a key factor in bacterial conjugation [83–85]. Elevated cell membrane permeability could be more favorable for plasmids mobilization between cells [83, 84]. In our study, biochar significantly reduced bacterial cell membrane permeability, which may be also linked to the decrease of conjugation transfer in manured soil.

Using sterilized manure, we have confirmed that the nonliving substances in manure significantly promoted the conjugative transfer of ARGs in soils. The nonliving substances in manure include heavy metals, antibiotics, salts, and nutrients. These contents in manure have been found to be associated with HGT, but their causation relationships have rarely been explored [19, 23]. By rinsing sterilized manure to remove some heavy metals, antibiotics, salts, and nutrients (N, P, and K), we found that the conjugative transfer ratio in rinsed-manure treatment soil microcosm was significantly reduced compared to that of nonrinsed manure treatment. By contrast, significant increases in conjugative transfer were observed when manure or pollutants (heavy metals and antibiotics) were added to the soil or water (Fig. 7). These results highlight that the content of heavy metals and antibiotics in the manure play an important role in conjugative transfer of ARGs. Our work confirmed that biochar significantly reduced the bioavailability of pollutants in manured soil, and alleviated the stress of pollutants on bacteria, which is beneficial for reducing conjugative transfer process. In the presence of pollutants, the application of biochar significant decreased the conjugative transfer ratio in both soil and water (Fig. 7). It demonstrates that the reduced conjugative transfer of ARGs is attributed to the decreased pollutant bioavailability in manure after the treatment of biochar. Biochar has a good adsorption capacity for various antibiotics and heavy metals [86], which supported the biochar-induced efficient reduction in the bioavailability of both heavy metals and antibiotics in soils and the following minimized selective pressure on bacteria.

In summary, this study offers the direct evidence that biochar enables to reduce the spread of ARGs in manured soil and proves a causal relationship between biochar and its reduction of HGT of ARGs in soils. Simultaneous application of biochar with manure in soil can effectively reduce the dissemination of ARGs, offering a promising solution to the land application of manures and organic fertilizers. Regarding the contrasting impact of short-term HGT prevention versus soil fertility, biochar could be beneficial for promoting soil microbial diversity in the long term and increased the content of bioavailable N, Olsen-P, and K in soil (Supplementary Table S2). Although biochar had a significant inhibitory effect on soil microbial metabolism related to indicators such as ATP, cell membrane permeability, and protease activity on the first day, these inhibitory effects gradually weakened over time and basically returned to the level without biochar application (Fig. S10). We acknowledge that it remains uncertain whether the observed reduction leads to ecologically meaningful impacts under field conditions. This warrants further quantitative assessment in future studies. However, we emphasize that such changes are potentially significant, considering that more than 9 billion tonnes of animal manure emission and most of them are applied to agricultural land globally each year [1, 75]. Given this scale, even a several-fold increase in the abundance of ARB in the environment could plausibly elevate the risk of human exposure. Additionally, reducing the conjugative transfer of ARGs may offer further ecological benefits, particularly in terms of the widespread use of biochar in agricultural practices. However, the only wheat straw biochar and one type of soil was used in this study, whereas the interaction between biochar and soil is very complex. Besides the effect on soil properties and microorganisms, the biochar mediated formation of ROS in soil may also have an impact on HGT of ARGs [87]. Therefore, it requires determining whether other types of biochar and soil have similar functions in further studies.

It is too complicated to study the ARG transfer pattern between manure microbiota and soil microbiota, as there are very diverse bacterial species existing in both manure and soil communities. To identify which indigenous bacteria in soils become transconjugants, this study simplified the conjugation system by choose one bacterial strain existing manure bacteria as a representative donor (i.e. a fluorescent labeled tracer *E. coli*). However, when considering the wide variety of bacteria co-existing in manure, it remains unknown how the dissemination dynamics of antibiotic resistance at a community-wide level, which should be further investigated in future studies.

## Supplementary Material

Supplementary_information_wraf187

## Data Availability

The metagenomic raw sequencing data generated in this study have been deposited in the National Center for Biotechnology Information (NCBI) SRA database (https://www.ncbi.nlm.nih.gov/sra/PRJNA1201867) under the BioProject PRJNA1201867.
