# Supplementary material for: Biochar suppresses conjugative transfer of antibiotic resistance genes in manure-amended soils
Source: ISME J. 2025 Aug 21;19(1):wraf187. doi: 10.1093/ismejo/wraf187 (PMC12422101; doi:10.1093/ismejo/wraf187)
Supplement: Supplementary_information_wraf187 [file supplementary_information_wraf187.pdf]

Supplementary information

Biochar suppresses conjugative transfer of antibiotic resistance genes in manure-amended soils

Jing Fang[1,2], Zhiwen Chen[1], Zhigang Yu[3], Shengdao Shan[1,2*], Yucheng Hou[1], Lili Liu[1], Jin Huang[4,5], Bing Li[4], Jianhua Guo[3*]

[1]School of Environment and Natural Resources, Zhejiang University of Science and Technology, Hangzhou 310023, China.

[2]Key Laboratory of Recycling and Eco-treatment of Waste Biomass of Zhejiang Province, Hangzhou 310023, China.

[3]Australian Centre for Water and Environmental Biotechnology, The University of Queensland, St. Lucia, Brisbane, QLD, Australia.

[4]Key Laboratory of Microorganism Application and Risk Control, Ministry of Ecology and Environment, Tsinghua Shenzhen International Graduate School, Tsinghua University, Shenzhen 518055, China.

[5]School of Minerals Processing and Bioengineering, Central South University, Changsha 410083, China.

[*]Corresponding authors

Corresponding authors:
Professor Shengdao Shan,
School of Environment and Natural Resources, Zhejiang University of Science and Technology, Hangzhou 310023, China
Email: shashd@vip.sina.com

Professor Jianhua Guo,
Australian Centre for Water and Environmental Biotechnology, The University of Queensland, St. Lucia, Brisbane, QLD 4072, Australia
Email: jianhua.guo@uq.edu.au

Text S1: Determination of the minimal inhibitory concentration (MIC) of the donor *E. coli.*

MICs of tetracycline (TC) and trimethoprim (TMP) for the donor *E. coli* were determined by the broth microdilution method as recommended by the references [1, 2]. Serial twofold dilutions of antibiotics were prepared in MuellerHinton broth, and 100 μL of 1×10^6 CFU/mL bacterial suspension was added to 100-μL antibiotic in each well and incubated at 37 °C for 18 h. MIC was observed using the resazurin test and recorded as the lowest concentration of the antibiotic without visible color change after 2 h of treatment.

Text S2: Characterizations of biochar samples

Total C, H, and N contents of biochar samples were measured using an elemental analyzer (MicroCube, Elementar, Germany) and the O content was determined by the mass balance calculation as previously reported [3]. The ash content was determined by a direct ash content method, which involved burning biochar samples in a muffle furnace at 750 °C for 4 hours under air and then calculating it using the differential weight method [4]. The Brunauer-Emmett-Teller (BET) surface area analysis was conducted on a Quantachrome, AUTO-SORB AS-1 surface area analyzer (Micromeritics Instrument Co. Ltd., USA). The pH of biochar in ultrapure water (1:50, m/v) was measured with pH meter. The H/C and O/C or (O+N)/C atomic ratios were calculated to evaluate the aromaticity and polarity of the biochar, respectively.

Text S3: Determination of nutrients in soil and manure

Available N, Olsen-P and Available K in soil and manure were measured using the alkaline hydrolysis diffusion method [5], Olsen method [6], and ammonium acetate leaching and flame photometry [7], respectively. The conductivity of soil and manure was measured by the ratio of 1:10 solid and water (w/w), and the suspension was filtered after homogenization, and the conductivity meter was used to measure the soil conductivity after standing for 2 hours.

Text S4: Set up method for soil microcosm series with non-sterilized manure

Briefly, they were prepared in 50 mL tubes by adding 15 g of soil (fresh soil), 0.15 g of biochar (about equal to 1% application rate), 0.6 g of non-sterilized manure (about equal to 40 mg/g soil) , around 9 mL ultrapure water in sequence. Before adding the next substance, the previous substances were evenly mixed. Finally the mixture was stirred just to form slurry and incubated at 30 °C in the

dark for up to 24 hours in an incubator. The tubes were opening to allow gas exchange through incubation. Soil microcosm was irrigated with ultrapure water to compensate for the weight loss due to evaporation during incubation by weighing method every 12 hours.

Text S5: Set up method for soil microcosm series with sterilized manure

In order to eliminate the influence of manure bacteria on the conjugation process, the manure was sterilized by autoclave before use, which was used as only nutrient source in the study. Soil microcosm series with sterilized manure were also prepared in 50 mL tubes by adding 15 g of soil (fresh soil), 0.15 g of biochar (about equal to 1% application rate), 0.6 g of sterilized manure (about equal to 40 mg/g soil), around 9 mL ultrapure water, and the donor bacteria in sequence. Before adding the next substance, the previous substances were evenly mixed. Finally, the mixture was stirred just to form slurry and incubated at 30 °C in the dark for up to 1 day, 7 days, and 30 days in an incubator, respectively. The tubes were opening to allow gas exchange through incubation. Soil microcosm was irrigated with ultrapure water to compensate for the weight loss due to evaporation during incubation by weighing method every 12 hours. The amount of manure used approximately corresponded to a general manure application for arable soils. The donor bacteria suspension was fully mixed with soil, providing a final donor strain density of about 1.3×10^7 CFU/g soil. It should be noted that the donor strain is the only plasmid donor that can trace the spread of ARGs in soil and the full set of plasmids/HGT/ARG potential in manure is excluded.

Text S6: Method for rinsing manure and the corresponding soil microcosms

In order to remove some heavy metals and antibiotics, the manure was rinsed. Briefly, two grams of sterilized manure were mixed into 40 mL of ultrapure water in a 50 mL centrifuge tube. The mixture was shaken on a shaker at 150 r/min for 2 hours, followed by centrifugation at 10, 000 r/min for 5 min. Then the supernatant extract was removed carefully and added 40 mL of ultrapure water again. The above cleaning process was repeated for three times and the final precipitate (the rinsed manure) was stored at 4 °C. Soil microcosm series with the rinsed manure were prepared in 50 mL tubes containing 15 g of soil (fresh soil), 0.15 g of biochar (about equal to 1% application rate), 0.6 g of the rinsed manure (about equal to 40 mg/g soil), around 9 mL ultrapure water and the donor bacteria in sequence. The mixture was stirred just to form slurry and incubated at 30 °C in the dark for 24 hours in an incubator. The tubes were opening to allow gas exchange through

incubation. Soil microcosm was irrigated with ultrapure water to compensate for the weight loss due to evaporation during incubation by weighing method every 12 hours. The donor bacteria suspension was fully mixed with soil, providing a final donor strain density of about 1.3×10^7 CFU/g soil. Soil microcosm with various biochars or rinsed manure application was labeled as R-manure, R-manure+BC300 and R-manure+BC700 treatment groups, respectively.

Text S7: Soil microcosms with tetracycline treatment

Soil microcosm with tetracycline treatment was prepared in 50 mL tubes by adding 15 g of soil (fresh soil), 0.15 g of biochar (about equal to 1% application rate), around 9 mL ultrapure water, 75 μL of 1mg/mL tetracycline solution (about equal to 5 mg/kg of tetracycline in soil) and the donor bacteria in sequence. The addition rate 5 mg/kg of tetracycline in soil was within the concentration range of antibiotics (μg/kg to a few mg/kg) detected in soils adjacent to feedlots [8,9]. The mixture was stirred just to form slurry and incubated at 30 °C in the dark for 24 hours in an incubator. The tubes were not tightly closed to allow gas exchange through incubation. The donor bacteria suspension was fully mixed with soil, providing a final donor strain density of about 1.3×10^7 CFU/g soil. Soil microcosm with various types of biochar or tetracycline was labeled as soil+TC, soil+TC+BC300 and soil+TC+BC700 treatment groups, respectively.

Text S8 Pure culture (PBS solution) system set up

Pure culture system was set up in PBS solution in 50 mL tubes by adding 19 mL PBS solution, 0.2 g sterilized manure, 0.02 g biochar, 0.5 mL donor bacteria fluid and 0.5 mL recipient bacteria in sequence. *Escherichia coli* MG1655 (chromosomally tagged by *lacI^q*-P*lpp-mCherry*) hosting the broad host range plasmid pKJK5 (IncP1; tagged with P*lac-gfpmut3B,* carrying trimethoprim, tetracycline and kanamycin resistance genes) was used as donor, while the recipient strain was *Escherichia coli* DH5α with pUC18 plasmid carrying *tetM* resistance gene. The final donor strain and recipient strain density of about 1×10^8 CFU/mL. In order to investigate the effects of the soluble pollutants of manure on the conjugation transfer ratio, we added antibiotics or heavy metals that were equivalent to manure to PBS solution instead of manure. The addition of tetracycline was 50 μg/L that was equivalent to the total soluble antibiotics of manure (0.2 g) in 20 mL PBS. The additions of heavy metals were Cu 50 μg/L + Zn 92 μg/L + Pb 11 μg/L + Cd 10 μg/L + Ni 18 μg/L in PBS. The mixture was incubated at 30 °C in the dark for 24 hours in an incubator. After then the

mixture was filtered with a 20 μm cell filter, and stored the filtrate at 4 °C before detection.

Text S9 Survival test of the donor and recipient strain in the presence of biochar

The *Escherichia coli* MG1655 (chromosomally tagged by lacIq-Plpp-mCherry) hosting the broad host range plasmid pKJK5 (IncP1; tagged with Plac-gfpmut3B, carrying trimethoprim, tetracycline and kanamycin resistance genes) was used as donor, while the recipient strain was *Escherichia coli* DH5α with pUC18 plasmid carrying *tetM* resistance gene. The donor strain was cultured in selective LB containing 5 mg/L TC and 30 mg/L TMP for 9 h, while the recipient strain was cultured in selective LB containing 5 mg/L TC for 9 h. After culturing in an incubator shaker at 37 °C, the recipients and donors were washed three times with PBS to wash out antibiotics in the medium. BC300 and BC700, 0.02g for each, were respectively mixed with 19.5 mL of phosphate-buffered saline (PBS) and either 0.5 mL donor bacterial suspension or 0.5 mL recipient bacterial suspension, with all components adding sequentially under sterile conditions. The final donor strain and recipient strain density of about 1×10^8 CFU/mL, respectively. The above mixtures were incubated at 37 °C with shaking at 250 rpm for 24 h. Each treatment was conducted in biological triplicates, and the system was incubated at 37°C. Post bacterial incubation was conducted by dilution spread plate method. Briefly, 1 mL aliquots underwent serial dilution in PBS. 100 μL from final dilutions were plated onto the agar plates. Plates were incubated at 37 °C for 12 h, after which colony-forming units were quantified to calculate survival rates.

Text S10 Flow cytometry analyses for transconjugants

The flow cytometer was equipped with 488 and 561 nm two-laser system. To detect bacterial cells, both forward scatter (FSC) and side scatter (SSC) were used, and their threshold was lowered to the minimum of 200 in signal height. The green fluorescence of GFP was excited by 488 nm laser and collected in bandpass Filter1 (530/30 nm). The red fluorescence of mCherry was excited by 561 nm and checked in bandpass Flilter2 (610/20 nm). The gating was made so that a double logarithmic bivariate plot with FSC-Area and SSC-Area was used to detect events in the bacterial size and complexity. Soil control bacteria community was prepared as a fluorescence negative control. Donor strain was prepared as mCherry dsRed-positive and GFP-negative control. Up to 1×10^6 events were quantified per measurement. According to the fluorescent intensity of control bacteria, the events with high green fluorescence intensity and no red fluorescence intensity was gated,

sorted and collected to a 5 ml BD Falcon collection tubes based on the signal of FSC, SSC, Filter 1 and Filter 2. Before being loaded, the samples were diluted in PBS until an event rate of ~3000 events/s was obtained.

Text S11: 16S rRNA gene amplicon sequencing and bioinformatics analysis

16S rRNA gene amplicon sequencing was conducted at Majorbio Testing Center (Shanghai Meiji Biomedical Technology Co., Ltd). In brief, the hypervariable region V3-V4 of the bacterial 16S rRNA gene were amplified with primer pairs 338F (5'-ACTCCTACGGGAGGCAGCAG-3') and 806R (5'-GGACTACHVGGGTWTCTAAT-3') byT100 Thermal Cycler PCR thermocycler (BIO-RAD, USA) [10]. The PCR reaction mixture including 4 μL 5 × Fast Pfu buffer, 2 μL 2.5 mM dNTPs, 0.8 μL each primer (5 μM), 0.4 μL Fast Pfu polymerase, 10 ng of template DNA, and ddH_2O to a final volume of 20 μL. PCR amplification cycling conditions were as follows: initial denaturation at 95℃ for 3 min, followed by 27 cycles of denaturing at 95℃ for 30 s, annealing at 55℃ for 30 s and extension at 72 ℃ for 45 s, and single extension at 72℃ for 10 min, and end at 4℃. The PCR product was extracted from 2% agarose gel and purified using the PCR Clean-Up Kit (YuHua, Shanghai, China) according to manufacturer's instructions and quantified using Qubit 4.0 (Thermo Fisher Scientific, USA). Purified amplicons were pooled in equimolar amounts and paired-end sequenced on an Illumina PE300/ PE250 platform (Illumina, San Diego, USA) according to the standard protocols by Majorbio Bio-Pharm Technology Co. Ltd. (Shanghai, China). The raw sequencing reads were deposited into the NCBI Sequence Read Archive (SRA) database (Accession Number PRJNA746678) and were analyzed using DADA2 pipeline to obtain amplicon sequence variants (ASVs) [11]. Data analysis was performed using phyloseq version 1.11.3 in R statistical software version 3.6.3 and RStudio (Version 1.2.5033; http://www.rstudio.com/). Two datasets were created based on the sample's origin. One consisted of the ASVs present in the soil microcosms, and the other of ASVs from the presumable transconjugant datasets. In the soil microcosm and transconjugant datasets, ASVs were removed that were not assigned to bacteria, and assigned to chloroplasts or mitochondria. Bioinformatic analysis of the soil/gut microbiota was carried out using the Majorbio Cloud platform (https://cloud.majorbio.com). Based on the OTUs information, rarefaction curves and alpha diversity indices including observed OTUs, Chao1 richness was calculated with Mothur v1.30.1 [11, 12].

Text S12: Determination of bioavailable antibiotics and heavy metals in soil and manure

According to the methods from U.S. Environmental Protection Agency (EPA) Method 1694: Pharmaceuticals and Personal Care Products in Water, Soil, Sediment, and Biosolids by HPLC/MS/MS, five antibiotics were detected in manure used in this study, with tetracycline, trimethoprim and sulphacetamide ranking the top three, and the other two having extremely low levels of content (lincomycin and sulfadimidine). Therefore, we quantitatively monitored the content of top three abundant antibiotics in soil microcosms. Five common heavy metals in manure were monitored including Cu, Zn, Pb, Cd and Ni. The content of bioavailable antibiotics in soil and manure was extracted by ultrapure water and determined by high performance liquid chromatography coupled with triple quadrupole tandem mass spectrometry (HPLC-MS-MS) after concentration [13]. Briefly, 35 g of soil or manure was added to 40 mL of ultrapure water and shaken for 6 hours and then filtered by 0.22 μm filters. The filtrate was collected and purified by C18 solid phase extraction column (HLB). Then it used acetone/methanol (1:1) to wash the HLB column and concentrated the elution by nitrogen blowing device.

The contents of bioavailable Cu, Zn, Pb, Cd and Ni in soil and manure were extracted by DTPA method [14]. Briefly, 15 g of soil or manure was added to 20 mL of DTPA extraction solution and shaken for 2 hours at 200 rpm at 25 °C. After then, the soil suspensions were filtered by 0.22 μm filters, and the filtrate was used to determine the heavy metals by inductively coupled plasma mass spectrometry (ICP-MS).

Table S1. Physical and chemical properties of biochar

Biochar	pH	Ash %	Surface areas $m^2\ g^{-1}$	Elements %				Atomic ratio		
				C	H	N	O	H/C	O/C	(O+N)/C
BC300	8.1	12.9	3.4	50.5	3.3	1.1	31.8	0.79	0.47	0.49
BC700	10.8	16.5	4.5	54.3	1.1	0.8	26.8	0.25	0.37	0.38

Table S2. Physical and chemical properties of manure and soil microcosm

Materials	pH	Conductivity μs cm^{-1}	Nutrients（mg kg^{-1}）		
			Olsen-P mg kg^{-1}	Available N mg kg^{-1}	Available K mg kg^{-1}
soil	5.5	51.8	5.9	41.9	57.1
manure	8.2	2940.1	839.9	2084.7	3509.2
rinsed manure	7.9	283.4	570.8	1250.3	307.4
soil +BC300	7.8	204.7	8.6	49.4	371.4
soil+BC700	8.3	279.4	7.3	46.1	497.7
soil + manure	6.9	254.8	77.3	110.0	285.6
soil +manure+BC300	7.2	466.2	112.3	117.9	511.1
soil +manure+BC700	7.7	477.5	91.9	141.6	608.1

Note: The amount of manure added to the soil is 40 mg/g, and the amount of biochar added to the soil is 1% (w/w).

Table S3. Bioavailable antibiotics in manure and rinsed manure (μg kg^{-1})

Materials	Tetracycline	Trimethoprim	Sulphacetamide	Total 3 antibiotics
manure	4233.8	602.1	1.1	4837.0
Rinsed manure	681.8	316.2	1.0	999.1

Note: The manured referred above was sterilized manure.

Table S4. Bioavailable heavy metals in manure and rinsed manure (mg kg^{-1})

Materials	Cu	Zn	Pb	Cd	Ni	Total 5 metals
manure	4.6	9.2	1.1	1.0	1.8	17.7
Rinsed manure	0.8	1.1	0.8	1.0	1.5	5.2

Note: The manured referred above was sterilized manure.

Table S5. Mean abundance of donor in soil microcosms after 1 and 7 days incubation, obtained by flow cytometry (1×10^6 cells were quantified per measure)

Treatment groups	1 day incubation	7 days incubation
soil	91945	42
soil+BC300	96777	16
soil+BC700	89667	15
soil+manure	77708	67
soil+manure+ BC300	83932	72
soil+manure+ BC700	82643	36

Note: The manured referred above was sterilized manure.

Table S6. Mean abundance of donor and transconjugant in soil microcosms after 30 days incubation, obtained by flow cytometry (1×10^6 cells were quantified per measure)

Treatment groups	Cell number of donor	Cell number of transconjugants
soil	1	0
soil+BC300	1	0
soil+BC700	2	0
soil+manure	1	0
soil+manure+ BC300	1	0
soil+manure+ BC700	1	0

Note: The manured referred above was sterilized manure.

Table S7. Relative abundance of microbial composition of transconjugant pools identified at both phylum and genus levels in manured soil microcosm with and without biochar after incubation for 1 day

Treatment group	Phylum (%)	genus	Genus level (%)
**soil+ manure**	***Pseudomonadota* (52.12)**	*Halomonas*	16.55
		* *Escherichia-Shigella*	4.94
		Pelagibacterium	4.55
		* *Klebsiella*	4.19
		Rhizobiaceae_unclassified	3.87
		Lysobacter	3.54
		Methylobacterium	3.51
		Oceanicaulis	2.87
		Ellin6055	1.34
		Enterobacteriaceae_unclassified	1.1
		Sphingomonadaceae_unclassified	0.68
		Parasutterella	0.67
		Morganella	0.63
		Dechloromonas	0.4
		Serratia	0.33
		Curvibacter	0.28
		* *Acinetobacter*	0.24
		Enhydrobacter	0.2
		Schlegelella	0.16
		Delftia	0.15
		Massilia	0.14
		Paracoccus	0.14
		Aliihoeflea	0.13
		Acidovorax	0.12
		Craurococcus	0.12
		Proteus	0.12
		Thermomonas	0.12
		Caulobacter	0.09
		Sutterella	0.09
		Rubellimicrobium	0.08
		Brevundimonas	0.07
		Hydrogenophilus	0.07
		Sphingomonas	0.07
		Hyphomonadaceae_unclassified	0.05
		* *Pseudomonas*	0.05
		Alkanindiges	0.04
		Qipengyuania	0.04
		Sphingobium	0.04
		Bilophila	0.03
		Burkholderiaceae_unclassified	0.03

		MN_122.2a	0.03
		Novosphingobium	0.03
		Ralstonia	0.03
		Ramlibacter	0.03
		Bosea	0.02
		Pajaroellobacter	0.02
		Actinobacillus	0.01
		AlphaPseudomonadota_unclassified	0.01
		Beijerinckiaceae_uncultured	0.01
		Bradyrhizobium	0.01
		Burkholderia-Caballeronia-Paraburkholderia	0.01
		Comamonas	0.01
		Defluviicoccus	0.01
		Desulfovibrio	0.01
		Desulfovibrionaceae_uncultured	0.01
		Devosiaceae_unclassified	0.01
		* *Haemophilus*	0.01
		Hafnia-Obesumbacterium	0.01
	Bacillota **（23.9）**	*Lachnospiraceae_unclassified*	2.69
		Faecalibacterium	2.02
		Veillonella	1.64
		Lachnospiraceae_ge	1.62
		Ammoniphilus	1.22
		Megamonas	1.12
		Streptococcus	0.95
		Ruminococcaceae_ge	0.88
		Subdoligranulum	0.78
		Blautia	0.74
		Lachnoclostridium	0.74
		Dialister	0.71
		Lactobacillus	0.6
		* *Staphylococcus*	0.6
		Agathobacter	0.55
		Lachnospira	0.51
		Brevibacillus	0.48
		Holdemanella	0.42
		Dorea	0.29
		Ruminococcaceae_UCG-014	0.28
		Ruminococcaceae_unclassified	0.26
		Clostridiales_unclassified	0.21
		Phascolarctobacterium	0.21
		Erysipelotrichaceae_UCG-003	0.19
		Ruminococcus_2	0.18

		Anoxybacillus	0.17
		Coprococcus_3	0.17
		Roseburia	0.16
		Ruminococcaceae_UCG-002	0.15
		Anaerostipes	0.14
		Megasphaera	0.14
		Romboutsia	0.14
		Butyricicoccus	0.13
		Flavonifractor	0.12
		Ruminococcaceae_UCG-013	0.12
		Ruminococcus_1	0.12
		* *Bacillus*	0.11
		* *Clostridium_sensu_stricto_1*	0.11
		Erysipelatoclostridium	0.11
		Fusicatenibacter	0.1
		Ruminiclostridium_5	0.1
		Lachnospiraceae_NK4A136_group	0.09
		Lachnospiraceae_UCG-004	0.09
		Paenibacillaceae_unclassified	0.09
		Lachnospiraceae_UCG-010	0.08
		Tyzzerella_3	0.08
		Lachnospiraceae_ND3007_group	0.07
		Lachnospiraceae_uncultured	0.07
		Anaerococcus	0.06
		Catenibacterium	0.06
		Mitsuokella	0.06
		Bacillales_unclassified	0.05
		Eisenbergiella	0.05
		Bacilli_unclassified	0.04
		CAG-56	0.04
		Ileibacterium	0.04
		Oscillibacter	0.04
		Ruminiclostridium_9	0.04
		Ruminococcaceae_UCG-004	0.04
		Salipaludibacillus	0.04
		Allisonella	0.03
		Anaerotruncus	0.03
		Christensenellaceae_unclassified	0.03
		Coprococcus_2	0.03
		Eubacterium	0.03
		Gemella	0.03
		Granulicatella	0.03
		Hungatella	0.03
		Intestinibacter	0.03

		Lachnospiraceae_FCS020_group	0.03
		Negativibacillus	0.03
		Peptoniphilus	0.03
		Ruminococcaceae_NK4A214_group	0.03
		UC5-1-2E3	0.03
		Vagococcus	0.03
		Atopostipes	0.02
		Bacillaceae_unclassified	0.02
		Faecalibaculum	0.02
		Lachnospiraceae_UCG-003	0.02
		Lysinibacillus	0.02
		Phocea	0.02
		Ruminiclostridium	0.02
		Ruminococcaceae_UCG-005	0.02
		Ruminococcaceae_UCG-010	0.02
		Tyzzerella	0.02
		UBA1819	0.02
		Weissella	0.02
		Abiotrophia	0.01
		Acidaminococcaceae_unclassified	0.01
		Aerococcaceae_unclassified	0.01
		Ammoniibacillus	0.01
		Catonella	0.01
		Chryseomicrobium	0.01
		* *Enterococcus*	0.01
		Facklamia	0.01
		Family_XIII_ge	0.01
		Family_XIII_UCG-001	0.01
		Bacillota_unclassified	0.01
		GCA-900066575	0.01
		Holdemania	0.01
		Lachnospiraceae_NK4A136_group	0.01
		Lachnospiraceae_UCG-001	0.01
		Limnochordaceae_ge	0.01
		Mogibacterium	0.01
	***Bacteroidota* (16.91)**	*Bacteroides*	9.74
		Prevotella_9	3.67
		Prevotella_2	1.1
		Parabacteroides	0.5
		Sediminibacterium	0.48
		Alistipes	0.41
		Muribaculaceae_ge	0.36
		Prevotellaceae_unclassified	0.16
		Paraprevotella	0.14

		Alloprevotella	0.07
		Bacteroidales_unclassified	0.04
		Barnesiella	0.03
		Butyricimonas	0.03
		Chryseobacterium	0.03
		Odoribacter	0.03
		Prevotella	0.02
		Prevotellaceae_NK3B31_group	0.02
		Prevotellaceae_UCG-001	0.02
		Bacteroidia_unclassified	0.01
		Capnocytophaga	0.01
		Cnuella	0.01
		Muribaculaceae_unclassified	0.01
		Porphyromonas	0.01
		Sphingobacterium	0.01
	Actinomycetota **（4.12）**	*Bifidobacterium*	1.17
		Nesterenkonia	0.97
		Microcella	0.39
		* *Corynebacterium_1*	0.28
		Collinsella	0.2
		Kocuria	0.18
		Frankiales_uncultured_ge	0.07
		Actinomyces	0.06
		Bifidobacteriaceae_unclassified	0.06
		Catenuloplanes	0.06
		Intrasporangiaceae_unclassified	0.06
		Micrococcus	0.06
		Brachybacterium	0.04
		Dietzia	0.04
		Eggerthella	0.04
		Rhodococcus	0.04
		Blastococcus	0.03
		Brevibacterium	0.03
		* *Cutibacterium*	0.03
		Gaiellales_uncultured_ge	0.03
		Marmoricola	0.03
		Micromonosporaceae_unclassified	0.03
		Acidothermus	0.02
		Atopobium	0.02
		Corynebacteriaceae_unclassified	0.02
		* *Corynebacterium*	0.02
		Gaiellales_unclassified	0.02
		Kytococcus	0.02
		Nocardioides	0.02

		Pseudonocardia	0.02
		Solirubrobacteraceae_unclassified	0.02
		Actinomycetaceae_unclassified	0.01
		Coriobacteriales_Incertae_Sedis_uncultured	0.01
		Dermacoccus	0.01
		Eggerthellaceae_unclassified	0.01
	Verrucomicrobiota **（2.26）**	*Akkermansia*	2.26
	Cyanobacteria **（0.18）**	*Nostocales_unclassified*	0.09
		Chroococcidiopsis_SAG_2023	0.07
		Obscuribacterales_ge	0.01
	Fusobacteriota **（0.14）**	*Fusobacterium*	0.13
		Leptotrichia	0.01
	Tenericutes **（0.09）**	*Mollicutes_RF39_ge*	0.09
	Gemmatimonadota **（0.07）**	*Gemmatirosa*	0.07
	Patescibacteria **（0.02）**	*Saccharimonadales_ge*	0.02
	Chloroflexota **（0.02）**	*JG30-KF-CM45_ge*	0.010
		KD4-96_ge	0.006
		Chloroflexota_unclassified	0.005
	Planctomycetes **（0.028）**	*Singulisphaera*	0.014
		Pla3_lineage_ge	0.008
		WD2101_soil_group_ge	0.006
	Bacteria_unclassified **（0.02）**	*Bacteria_unclassified*	0.02
	Deinococcus-Thermus **（0.009）**	*Deinococcus*	0.006
		Truepera	0.003
	WPS-2 **(0.005)**	*WPS-2_ge*	0.005
	**Others（0.038）**	Others	0.038
**soil+ manure+ BC300**	***Pseudomonadota*** **（56.45）**	*Delftia*	11.33
		Methylobacterium	6.72
		* *Roseomonas*	5.79
		Silanimonas	5.54
		norank_f__norank_o__norank_c__AlphaPseudomonadota	5.50
		unclassified_f__Enterobacteriaceae	4.86
		Bosea	4.69
		Ellin6067	2.37
		Massilia	2.16

		Sphingomonas	1.65
		Methyloversatilis	1.31
		Sphingobium	1.10
		Paracoccus	0.85
		unclassified_f__Hyphomicrobiaceae	0.68
		norank_f__A0839	0.51
		Pseudoxanthomonas	0.42
		Aureimonas	0.42
		norank_f__Hydrogenophilaceae	0.34
		* *Klebsiella*	0.21
	Bacillota **（26.98）**	*Ammoniphilus*	8.25
		* *Staphylococcus*	6.72
		Streptococcus	5.07
		Finegoldia	2.88
		* *Bacillus*	1.14
		Solibacillus	1.06
		Brevibacillus	0.76
		Fenollaria	0.59
		Lysinibacillus	0.51
	Actinomycetota **（10.44）**	* *Corynebacterium*	8.12
		Micrococcus	1.06
		Promicromonospora	0.42
		Kocuria	0.34
		Dietzia	0.17
		* *Cutibacterium*	0.17
		Brachybacterium	0.08
		Nocardioides	0.08
	Bacteroidota ***（0.89）***	*Prevotella*	0.89
	unclassified Bacteria **（4.94）**	*unclassified_k__norank_d__Bacteria*	4.94
	Cyanobacteria **（0.30）**	*Chroococcidiopsis_SAG_2023*	0.30
**soil+ manure+ BC700**	***Pseudomonadota*** **（59.32）**	*Massilia*	15.73
		Methylobacterium	8.37
		* *Escherichia-Shigella*	7.36
		* *Pseudomonas*	4.19
		Bosea	2.88
		Caulobacter	2.20
		* *Roseomonas*	1.61
		unclassified_f__Enterobacteriaceae	1.48
		Silanimonas	1.31

		Sphingobium	1.23
		Pantoea	1.14
		Delftia	1.14
		Methyloversatilis	1.06
		norank_f__Micavibrionaceae	0.97
		Niveispirillum	0.93
		* *Legionella*	0.89
		unclassified_f__Sphingomonadaceae	0.85
		Craurococcus-Caldovatus	0.63
		Acinetobacter	0.63
		Allorhizobium-Neorhizobium-Pararhizobium-Rhizobium	0.63
		Limnobacter	0.59
		Curvibacter	0.59
		Idiomarina	0.47
		Ralstonia	0.42
		unclassified_f__Alcaligenaceae	0.42
		revundimonas	0.38
		Rhodoplanes	0.30
		Roseobacter_clade_CHAB-I-5_lineage	0.25
		Qipengyuania	0.17
		* *Klebsiella*	0.13
		Dechloromonas	0.13
		Ancylobacter	0.08
		Sphingomonas	0.08
		norank_f__B1-7BS	0.04
		Enhydrobacter	0.04
	Bacillota **（16.54）**	*Ammoniphilus*	9.34
		* *Bacillus*	1.61
		Exiguobacterium	1.23
		Lactococcus	0.80
		* *Staphylococcus*	0.68
		Terribacillus	0.55
		Peptoniphilus	0.51
		Ureibacillus	0.51
		Lysinibacillus	0.47
		Anoxybacillus	0.42
		Laceyella	0.34
		Ezakiella	0.04
		Streptococcus	0.04
	Actinomycetota **（15.22）**	* *Corynebacterium*	8.75
		Micrococcus	1.82
		Pseudoclavibacter	1.14

		Dermacoccus	0.55
		Solirubrobacter	0.51
		Brachybacterium	0.47
		Kocuria	0.47
		Saccharopolyspora	0.34
		CL500-29_marine_group	0.25
		Prauserella	0.21
		Conexibacter	0.17
		Tetrasphaera	0.13
		Actinomyces	0.13
		* *Cutibacterium*	0.08
		* *Mycobacterium*	0.08
		Nocardioides	0.04
		Pseudonocardia	0.04
		norank_f__norank_o__Gaiellales	0.04
	***Bacteroidota* (4.96)**	*Chryseobacterium*	1.40
		Bacteroides	0.89
		norank_f__NS9_marine_group	0.51
		Empedobacter	0.47
		Niabella	0.38
		norank_f__Cyclobacteriaceae	0.38
		norank_f__Chitinophagaceae	0.34
		Xanthomarina	0.21
		norank_f__norank_o__norank_c__SJA-28	0.21
		norank_f__Microscillaceae	0.13
		norank_f__AKYH767	0.04
	***Cyanobacteria* (1.48)**	*unclassified_f__Chroococcidiopsaceae*	0.97
		Aliterella	0.42
		norank_f__unclassified_o__Oxyphotobacteria_Incertae_Sedis	0.04
		Nostoc_PCC-73102	0.04
	***Planctomycetes* (0.72)**	*norank_f__Tepidisphaeraceae*	0.51
		Tepidisphaera	0.21
	***Chloroflexota* (0.63)**	*norank_f__JG30-KF-CM45*	0.63
	***Verrucomicrobiota* (0.55)**	*Candidatus_Xiphinematobacter*	0.55
	***Fusobacteriota* (0.25)**	*Leptotrichia*	0.25
	***Acidobacteriota* (0.21)**	*norank_f__norank_o__11-24*	0.21
	***Bdellovibrionota* (0.04)**	*Bdellovibrio*	0.04

	Nitrospirota **（0.04）**	*Nitrospira*	0.04
	Patescibacteria **（0.04）**	*norank_f__norank_o__Candidatus _Moranbacteria*	0.04

Note: The manured referred above was sterilized manure. The genera marked with * refer to the bacteria species of pathogens and opportunistic pathogens.

Table S8. The top 10 bacterial genera in manured soil microcosms after incubation for 1 day

Soil microcosm	Genus and level	Phylum
soil+ manure	*Lysinibacillus*（46.74%）	*Bacillota*
	Solibacillus（29.40%）	*Bacillota*
	Kurthia（8.98%）	*Bacillota*
	Bacillus（8.36%）	*Bacillota*
	Acinetobacter（1.47%）	*Pseudomonadota*
	Brevibacillus（1.16%）	*Bacillota*
	Paenibacillus（0.62%）	*Bacillota*
	unclassified_c__Bacilli（0.34%）	*Bacillota*
	norank_f__Planococcaceae（0.28%）	*Bacillota*
	Aneurinibacillus（0.23%）	*Bacillota*
soil+ manure+ BC300	*Solibacillus*（21.57%）	*Bacillota*
	Lysinibacillus（20.02%）	*Bacillota*
	Bacillus（16.06%）	*Bacillota*
	Kurthia（15.17%）	*Bacillota*
	unclassified_f__Enterobacteriaceae（10.92%）	*Pseudomonadota*
	Acinetobacter（7.69%）	*Pseudomonadota*
	Pseudomonas（1.47%）	*Pseudomonadota*
	Brevibacillus（0.80%）	*Bacillota*
	Aneurinibacillus（0.74%）	*Bacillota*
	Paenibacillus（0.71%）	*Bacillota*
soil+ manure+ BC700	*Acinetobacter*（47.91%）	*Pseudomonadota*
	Solibacillus（28.32%）	*Bacillota*
	Lysinibacillus（8.30%）	*Bacillota*
	Bacillus（7.89%）	*Bacillota*
	Herbaspirillum（2.40%）	*Pseudomonadota*
	Kurthia（1.15%）	*Bacillota*
	Delftia（0.90%）	*Pseudomonadota*
	Aneurinibacillus（0.38%）	*Bacillota*
	norank_f__Planococcaceae（0.33%）	*Bacillota*
	Paenibacillus（0.32%）	*Bacillota*

Note: The manured referred above was sterilized manure.

Table S9. The top 10 genera bacteria in manured soil microcosms after incubation for 7 days

Soil microcosm	Genus and level	Phylum
soil+ manure	*Acinetobacter*（28.08%）	*Pseudomonadota*
	Ruminiclostridium（8.36%）	*Bacillota*
	Azospirillum（7.79%）	*Pseudomonadota*
	Lysinibacillus（5.00%）	*Bacillota*
	Bacillus（4.92%）	*Bacillota*
	Clostridium_sensu_stricto_8（4.62%）	*Bacillota*
	Delftia（4.30%）	*Pseudomonadota*
	Herbaspirillum（3.28%）	*Pseudomonadota*
	Clostridium_sensu_stricto_10（3.16%）	*Bacillota*
	Rhodococcus（2.88%）	*Actinobacteriota*
soil+ manure+ BC300	*Acinetobacter*（41.33%）	*Pseudomonadota*
	Delftia（17.13%）	*Pseudomonadota*
	Ruminiclostridium（5.31%）	*Bacillota*
	Herbinix（4.31%）	*Bacillota*
	Brevundimonas（4.17%）	*Pseudomonadota*
	Rhodococcus（2.91%）	*Actinobacteriota*
	Bacillus（2.80%）	*Bacillota*
	Clostridium_sensu_stricto_8（2.62%）	*Bacillota*
	Clostridium_sensu_stricto_10（1.45%）	*Bacillota*
	Azospirillum（1.23%）	*Pseudomonadota*
soil+manure+ BC700	*Acinetobacter*（41.57%）	*Pseudomonadota*
	Delftia（9.46%）	*Pseudomonadota*
	Herbinix（6.71%）	*Bacillota*
	Clostridium_sensu_stricto_8（5.63%）	*Bacillota*
	Ruminiclostridium（4.40%）	*Bacillota*
	Bacillus（2.99%）	*Bacillota*
	Sporomusa（2.77%）	*Bacillota*
	Solibacillus（2.58%）	*Bacillota*
	Chryseobacterium（2.22%）	*Bacteroidota*
	Azospirillum（1.90%）	*Pseudomonadota*

Note: The manured referred above was sterilized manure.

Table S10. The top 10 bacterial genera in manured soil microcosms after incubation for 30 days

Soil microcosm	Genus and level	Phylum
soil+ manure	*Bacillus*（10.80%）	*Bacillota*
	Acinetobacter（10.57%）	*Pseudomonadota*
	Rhodococcus（9.95%）	*Actinobacteriota*
	Azospirillum（7.49%）	*Pseudomonadota*
	Sinomonas（6.44%）	*Actinobacteriota*
	Ruminiclostridium（5.97%）	*Bacillota*
	Noviherbaspirillum（3.42%）	*Pseudomonadota*
	Massilia（3.07%）	*Pseudomonadota*
	Clostridium_sensu_stricto_10（3.01%）	*Bacillota*
	Clostridium_sensu_stricto_8（2.92%）	*Bacillota*
soil+ manure+ BC300	*Acinetobacter*（29.18%）	*Pseudomonadota*
	Bacillus（8.45%）	*Bacillota*
	Azospirillum（7.00%）	*Pseudomonadota*
	Rhodococcus（5.08%）	*Actinobacteriota*
	Ruminiclostridium（4.42%）	*Bacillota*
	Massilia（3.68%）	*Pseudomonadota*
	Sinomonas（3.59%）	*Actinobacteriota*
	Clostridium_sensu_stricto_10（3.02%）	*Bacillota*
	Clostridium_sensu_stricto_8（2.39%）	*Bacillota*
	Terrabacter（2.23%）	*Actinobacteriota*
soil+ manure+ BC700	*Azospirillum*（9.51%）	*Pseudomonadota*
	Ruminiclostridium（7.44%）	*Bacillota*
	Acinetobacter（7.07%）	*Pseudomonadota*
	Clostridium_sensu_stricto_8（4.48%）	*Bacillota*
	Pseudobacteroides（4.32%）	*Bacillota*
	norank_f__Halobacteroidaceae（4.29%）	*Halanaerobiaeota*
	Clostridium_sensu_stricto_10（3.66%）	*Bacillota*
	Methylobacterium-Methylorubrum（3.55%）	*Pseudomonadota*
	Herbinix（3.08%）	*Bacillota*
	Christensenellaceae_R-7_group（2.98%）	*Bacillota*

Note: The manured referred above was sterilized manure.

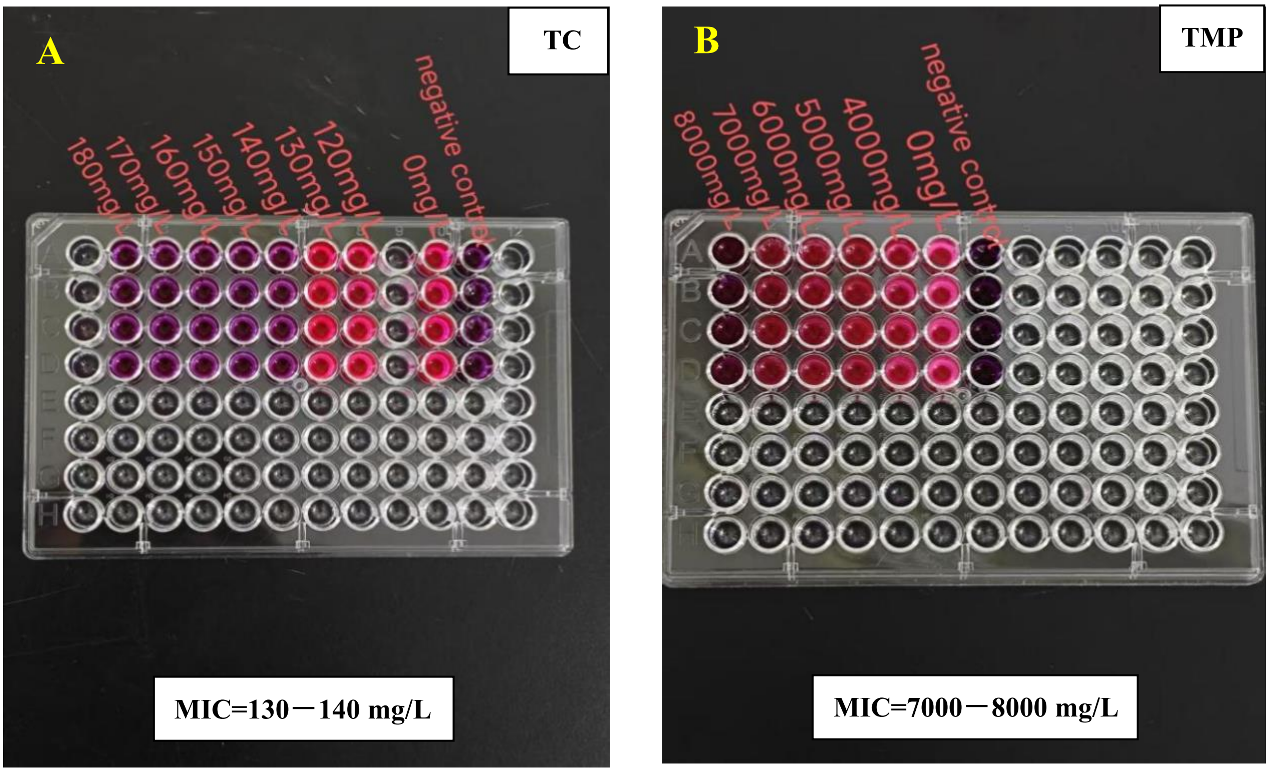

Fig. S1 Color changes of resazurin in the sensitivity test of tetracycline (TC) and trimethoprim (TMP).

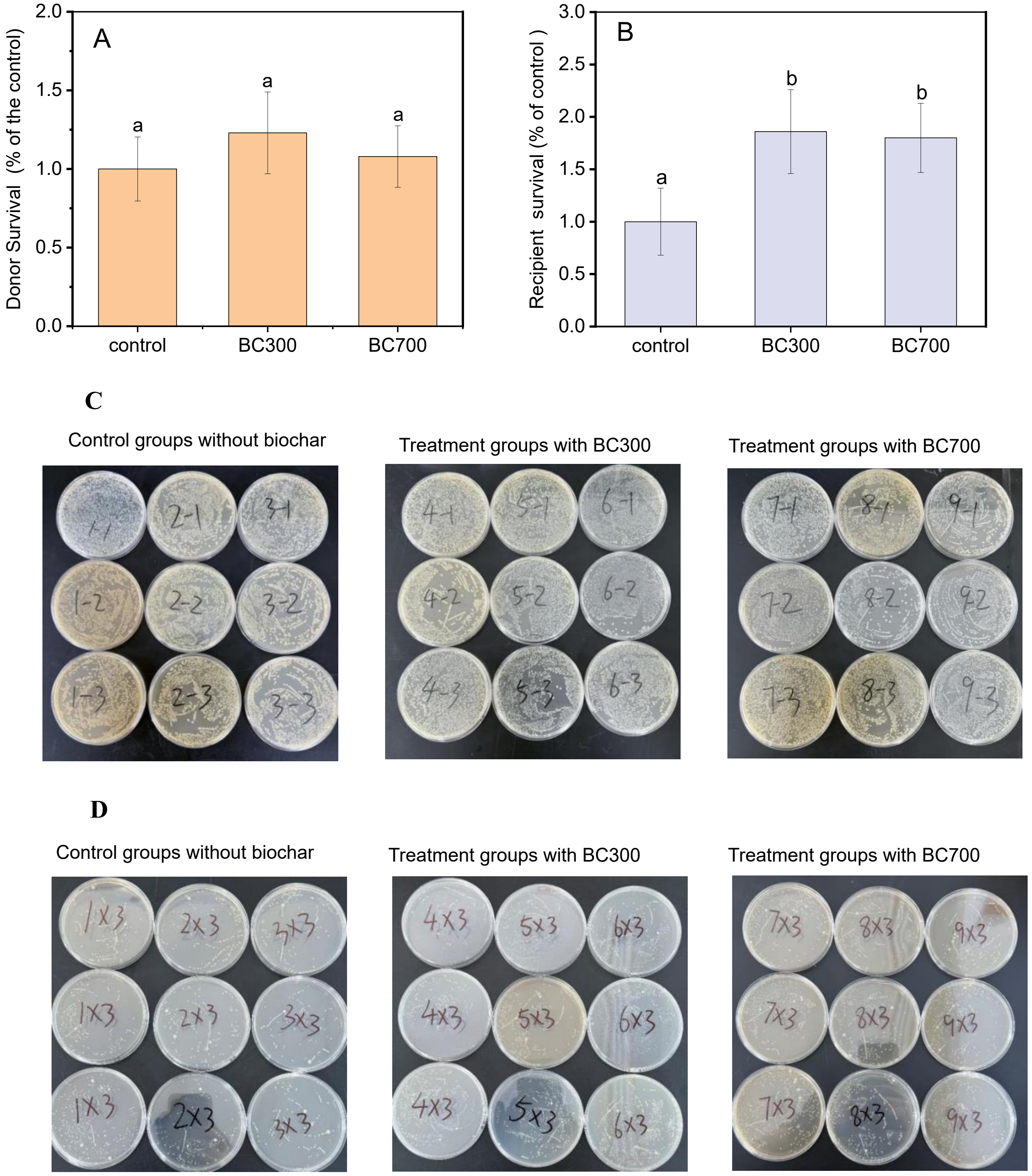

Fig. S2 The effect of biochar on E. coli donor/recipient viability. (a) Donor strain, (b) Recipient strain, (c) the experimental photos of donor, (d) the experimental photos of recipient. For each group, there were three biology replicates and three testing replicates. The different lowercase letters represent significant differences between treatment groups at the level of $P < 0.05$.

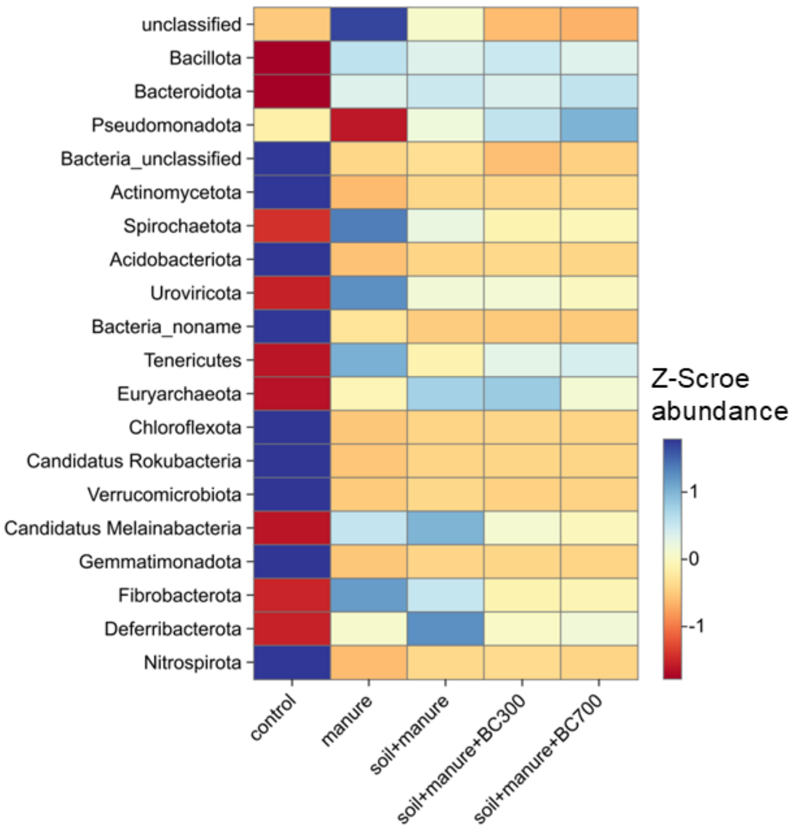

Fig. S3 Microbial community composition at phylum level (Top 20) in soil microcosms with different treatments after one-day incubation. The manure referred above was non-sterilized manure.

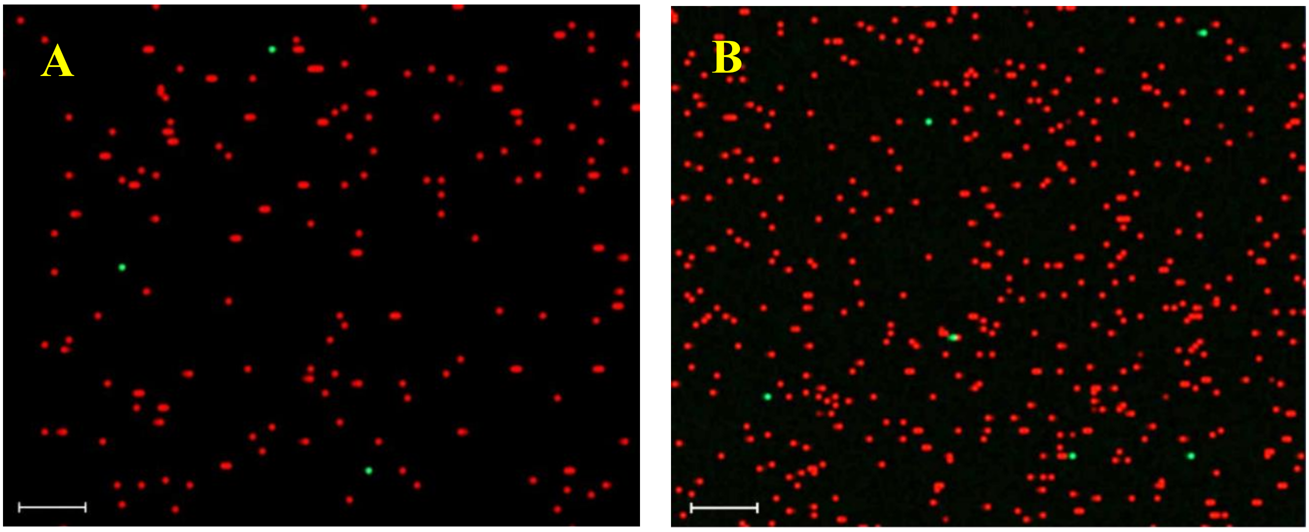

Fig. S4 Confocal laser scanning microscope (CLSM) images of the donor bacteria (red fluorescence) and transconjugants (green fluorescence) in soil microcosms after incubation for 1 day (A: soil+BC300, B: soil+sterilized manure + BC300). The original CLSM images were enhanced and adjusted for color contrast by ZEISS ZEN 3.12 application program. Scale bars = 8.5 µm.

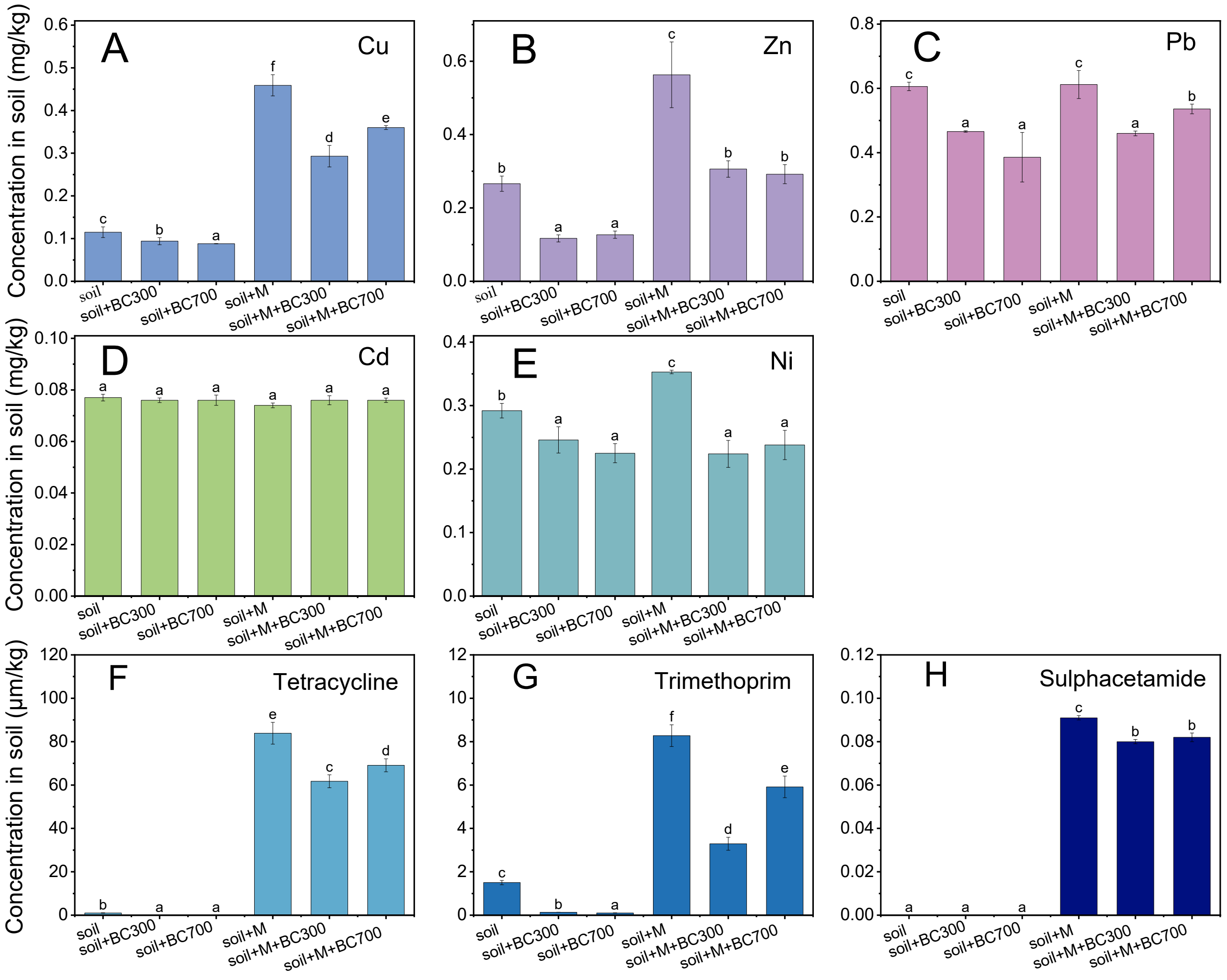

Fig. S5 The bioavailable heavy metals (A-E) and antibiotics (F-H) in soil microcosms after incubation for 1 day. A: Cu, B: Zn, c: Pb, D: Cd, E: Ni, F: Tetracycline, G: Trimethoprim, H: Sulphacetamide. M represents the sterilized manure. In each subplot, different lowercase letters represent significant differences between treatment groups at the level of $P < 0.05$.

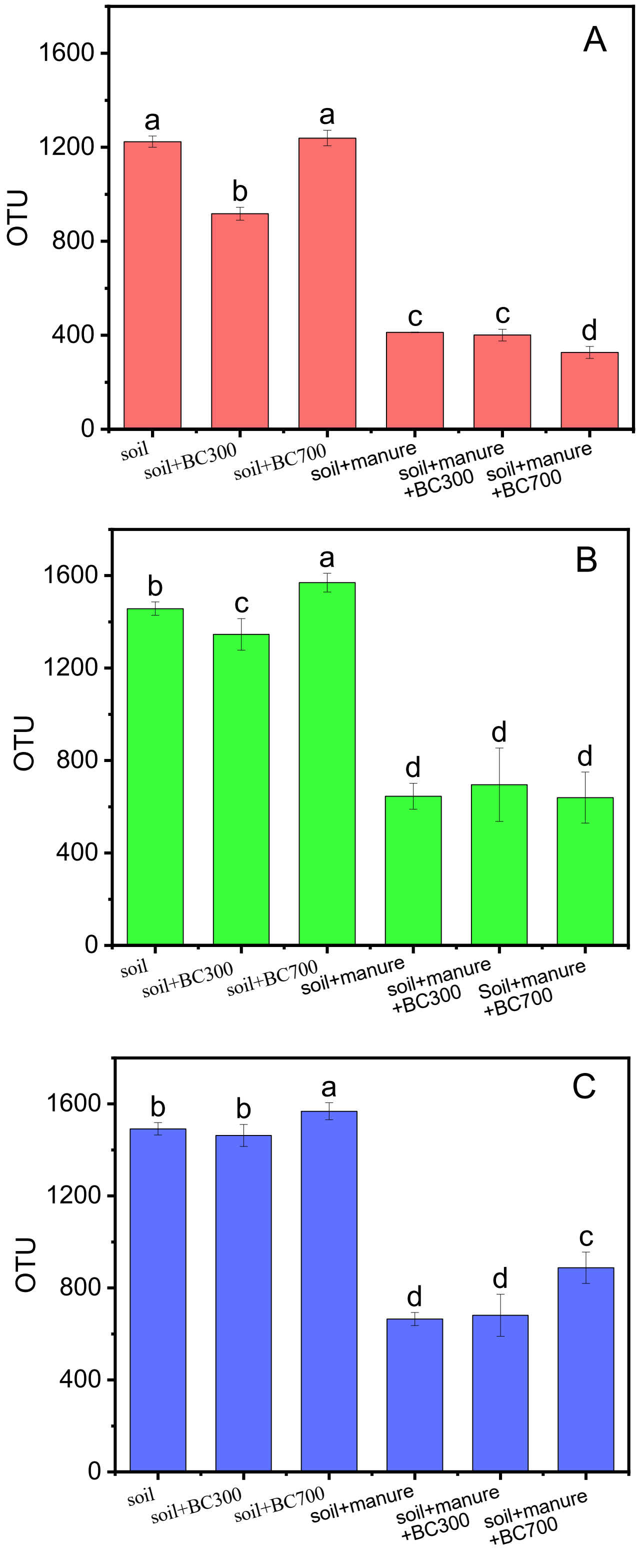

Fig. S6 The numbers of OUT in soil microcosms after incubation for 1 day (A), 7 days (B) and 30 days (C). In each subplot, different lowercase letters represent significant differences between treatment groups at the level of $P < 0.05$. The manure referred above was sterilized manure.

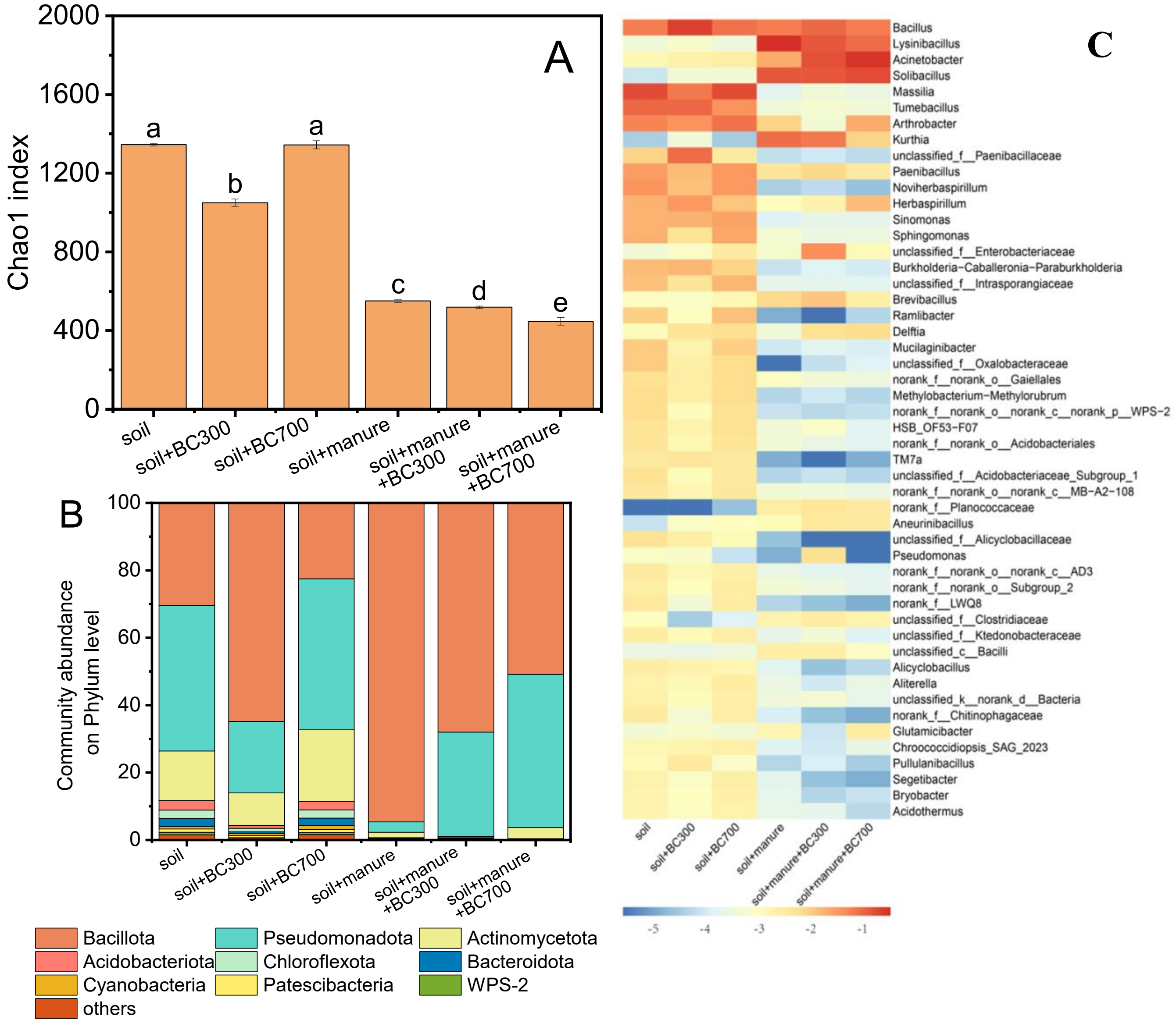

Fig. S7 Microbial diversity and community structure in soil microcosms with different treatments after incubation for 1 day. A：Chao1 index of soil bacteria; B: Relative abundance of various bacteria at phylum level; C: Relative abundance of various bacteria at genus level. In subplot A, different lowercase letters represent significant differences between treatment groups at the level of $P < 0.05$. The manure referred above was sterilized manure.

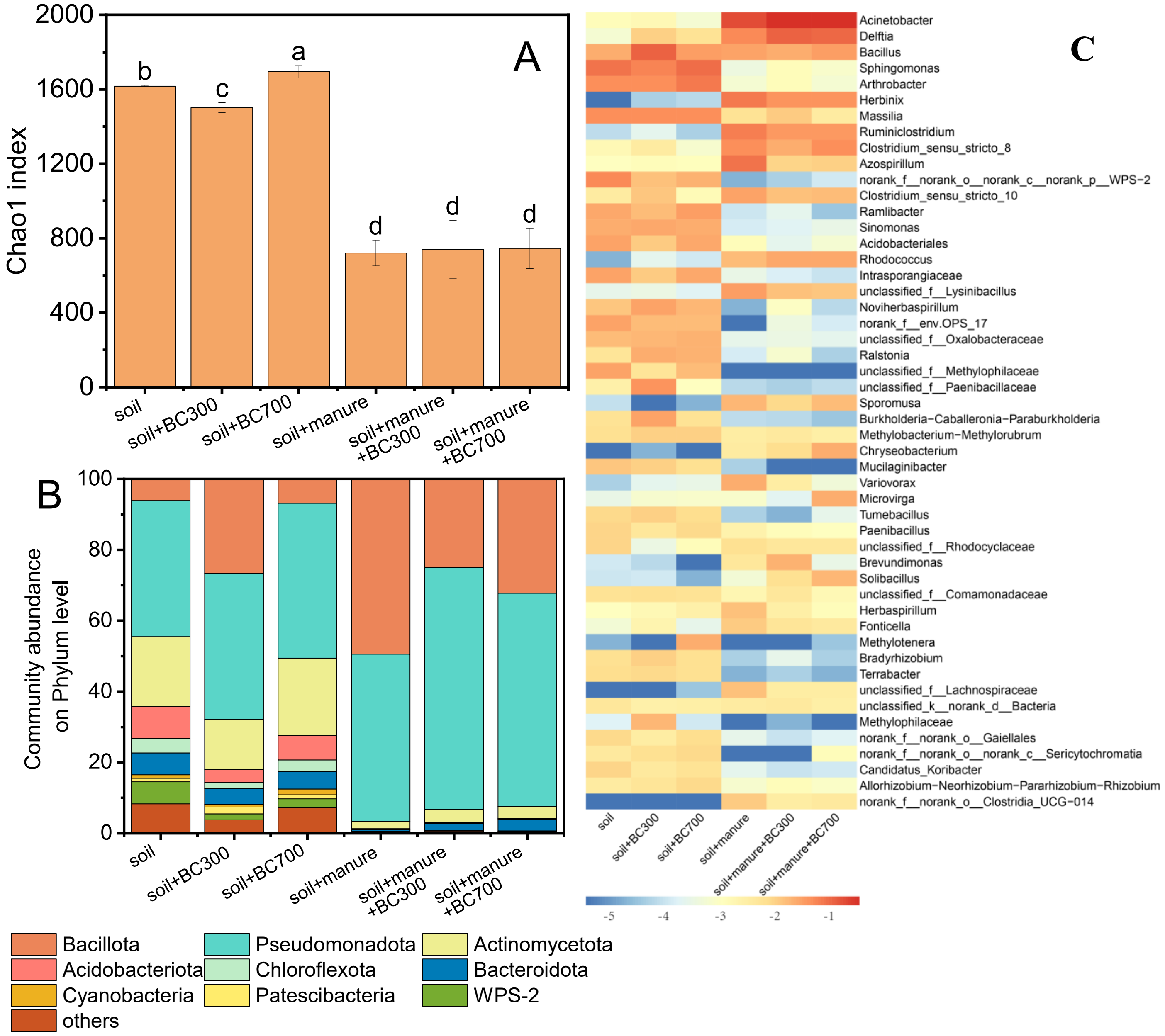

Fig. S8 Bacterial diversity and community structure in soil microcosms with different treatments after incubation for 7 days. A: Chao1 index of soil bacteria; B: Relative abundance at Phylum level; C: Relative abundance on genus level. In subplot A, different lowercase letters represent significant differences between treatment groups at the level of $P < 0.05$. The manure referred above was sterilized manure.

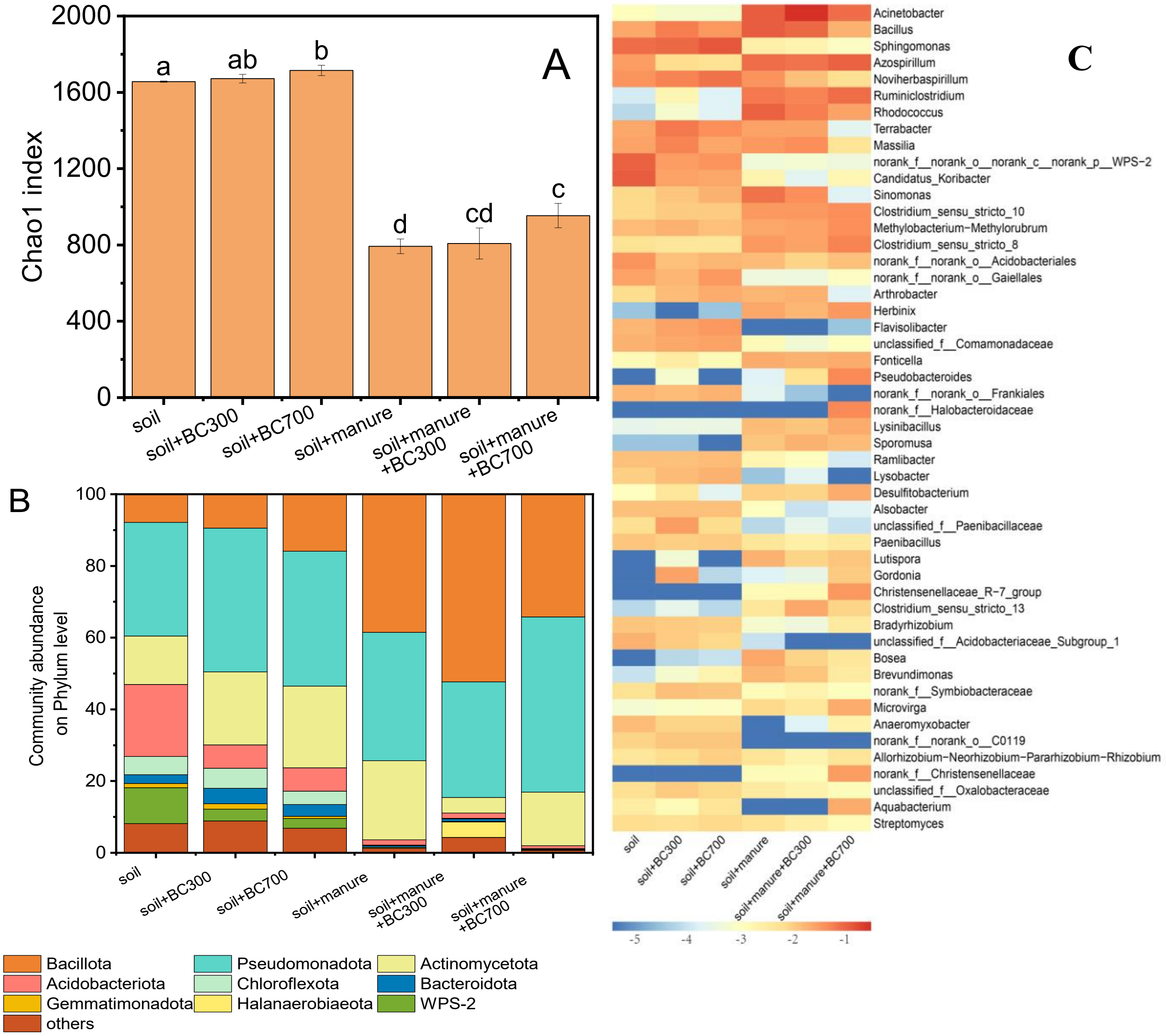

Fig. S9 Bacterial diversity and community structure in soil microcosms with different treatments after incubation for 30 days. A: Chao1 index of soil bacteria; B: Relative abundance at Phylum level; C: Relative abundance at genus level. In subplot A, different lowercase letters represent significant differences between treatment groups at the level of *P* < 0.05. The manure referred above was sterilized manure.

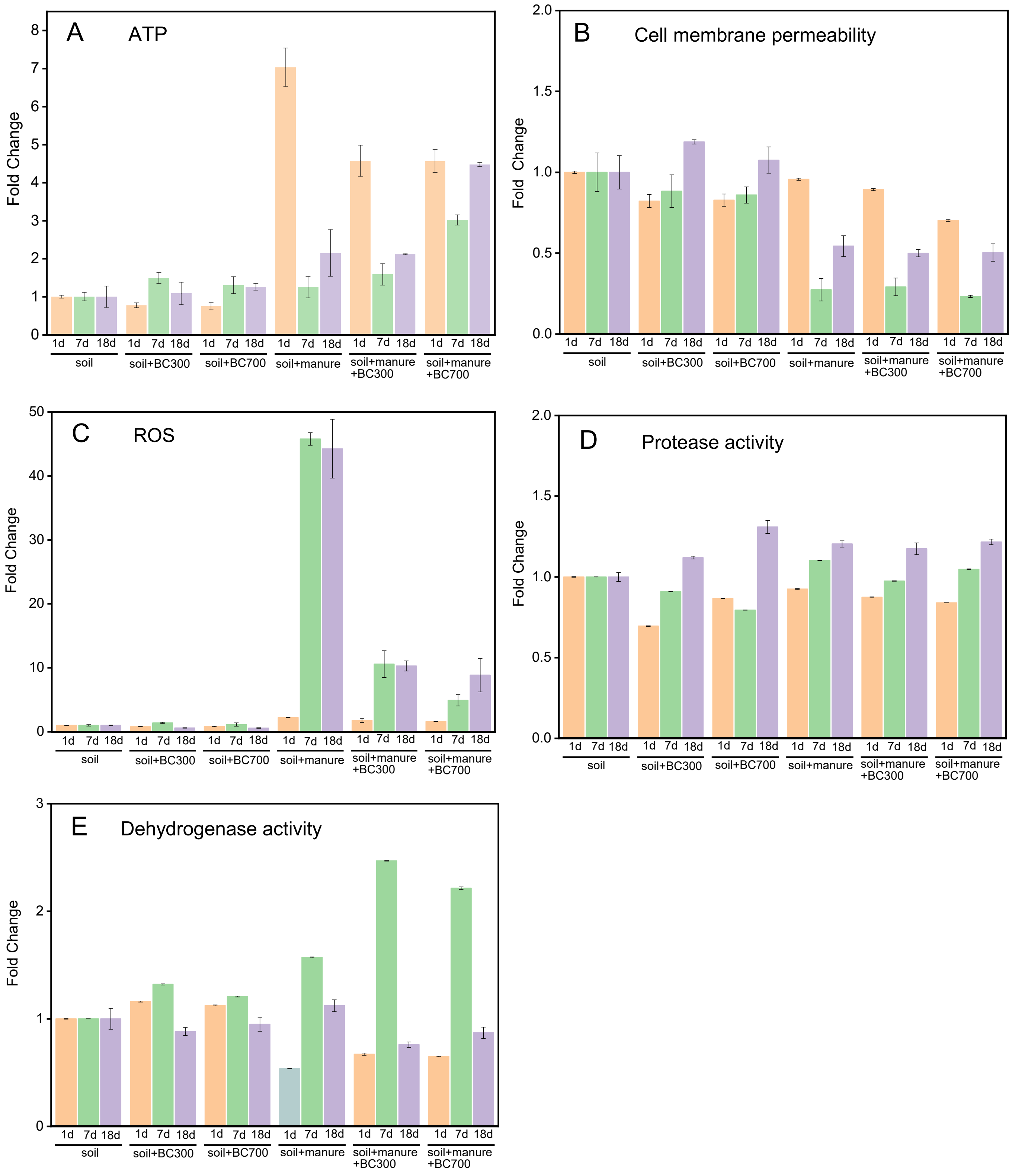

Fig. S10 The time frame of the effect of biochar on ATP, ROS and cell membrane permeability of soil bacteria as well as soil enzymes after cultivation for 1, 7 and 18 days. The manure referred above was sterilized manure. All the values are expressed as fold changes to the control soil group.

References

1. CLSI. 2020. Performance standards for antimicrobial susceptibility testing. 30th ed. Vol. CLSI supplement M100. Clinical and Laboratory Standards Institute, Wayne, PA.

2. Nwabor OF, Chukamnerd A, Terbtothakun P, Nwabor LC, Surachat K, Roytrakul S, et al. Synergistic effects of polymyxin and vancomycin combinations on carbapenem- and polymyxin-resistant Klebsiella pneumoniae and their molecular characteristics. Microbiology Spectrum. 2023;11: No 6. https://doi.org/10.1128/spectrum.01199-23.

3. Fang J, Cheng LL, Jin L, Wang DJ, Owens G, Lin DH. Release and stability of water dispersible biochar colloids in aquatic environments: Effects of pyrolysis temperature, particle size, and solution chemistry. Environ Pollut. 2020; 260: 114037.

4. Zheng H, Wang Z, Zhao J, Herbert S, Xing B. Sorption of antibiotic sulfamethoxazole varies with biochars produced at different temperatures. Environ Pollut. 2013;181: 60 – 67.

5. Lu RK. Analytical Methods of Soil Agricultural Chemistry. China Agricultural Science and Technology Press, Beijing, 2000. (in Chinese).

6. Olsen S R. Estimation of Available Phosphorus in Soils by Extraction with Sodium Bicarbonate (No. 939). US Department of Agriculture, USA, 1954.

7. Pages A L, Miller R H, Dennis R K. Methods of Soil Analysis. Part 2 Chemical Methods. Soil Science Society of America Inc., Madison, 1982.

8. Lyu, J., Yang, L., Zhang, L., Ye, B., & Wang, L. (2020). Antibiotics in soil and water in China – a systematic review and source analysis. Environmental Pollution, 266, 115147.

9. Qiao M., Ying G.G., Singer A.C., Zhu Y.G. Review of antibiotic resistance in China and its environment[J]. Environment International, 2018, 110,160-172.

10. Li L, Dechesne A, Madsen JS, Nesme J, Sørensen SJ, Smets BF. Plasmids persist in a microbial community by providing fitness benefit to multiple phylotypes. ISME J. 2020; 14: 1170–1181.

11. Edgar Robert C. UPARSE: highly accurate OTU sequences from microbial amplicon reads. Nature Methods, 2013; 10(10): 996–998.

12. Barberan A, Bates ST, Casamayor EO, Fierer N. Using network analysis to explore co-occurrence patterns in soil microbial communities. ISME J. 2012; 6: 343–351.

13. Wang Y, Dong X, Zang J, Zhao X, Jiang F, Jiang L, et al. Antibiotic residues of drinking-water and its human exposure risk assessment in rural Eastern China. Water Res. 2023; 236: 119940.

14. Wan YN, Huang QQ, Wang Q, Yu Y, Su DC, Qiao YH, et al. Accumulation and bioavailability of heavy metals in an acid soil and their uptake by paddy rice under continuous application of chicken and swine manure. J Hazard Mater. 2020; 384: 121293.
